# Structuring total angular momentum of light along the propagation direction with polarization-controlled meta-optics

**DOI:** 10.1038/s41467-021-26253-4

**Published:** 2021-10-29

**Authors:** Ahmed H. Dorrah, Noah A. Rubin, Michele Tamagnone, Aun Zaidi, Federico Capasso

**Affiliations:** 1 Harvard John A. Paulson School of Engineering and Applied Sciences, Harvard University, Cambridge, MA 02138 USA; 2 Fondazione Istituto Italiano di Tecnologia, Genova, Italy

**Keywords:** Applied optics, Metamaterials, Nanophotonics and plasmonics, Sub-wavelength optics

## Abstract

Recent advances in wavefront shaping have enabled complex classes of Structured Light which carry spin and orbital angular momentum, offering new tools for light-matter interaction, communications, and imaging. Controlling both components of angular momentum along the propagation direction can potentially extend such applications to 3D. However, beams of this kind have previously been realized using bench-top setups, requiring multiple interaction with light of a fixed input polarization, thus impeding their widespread applications. Here, we introduce two classes of metasurfaces that lift these constraints, namely: i) polarization-switchable plates that couple any pair of orthogonal polarizations to two vortices in which the magnitude and/or sense of vorticity vary locally with propagation, and ii) versatile plates that can structure both components of angular momentum, spin and orbital, independently, along the optical path while operating on incident light of any polarization. Compact and integrated devices of this type can advance light-matter interaction and imaging and may enable applications that are not accessible via other wavefront shaping tools.

## Abstract (web summary)

Creating complex forms of structured light typically requires bulky optics and multiple interactions with incident light. Here the authors demonstrate versatile control over light’s polarization and orbital angular momentum along the propagation direction with a single metasurface.

## Introduction

In addition to its linear momentum, light may carry angular momentum that manifests in two forms: spin and orbital^1,2^. Spin angular momentum (SAM) arises when the electric field vector traces a helical path with propagation, and takes the values of ±*ℏ* per photon, depending on the polarization handedness (i.e., right- or left-hand circular polarization)^3,4^. Orbital angular momentum (OAM), on the other hand, arises if the wavefront carries a phase singularity^5^. This is typically realized when the wavefront acquires a helical form, proportional to *e*^i*ℓϕ*^, producing a one-dimensional (1D) phase singularity—a line of undefined phase (and zero intensity) along the optical path^6^. In this case, the Poynting vector precesses around the phase singularity^7^, thereby producing a donut-like intensity profile, also known as an optical vortex. The helicity (vorticity) of the vortex beam is characterized by its topological charge value *ℓ*, which signifies the number of 2π phase twists acquired by the wavefront’s azimuth along a path equal to one wavelength. Unlike SAM which can only exhibit two states, or a weighted average of the two, OAM can possibly exhibit unbounded values per photon^8^. For paraxial beams, SAM and OAM are additive so that the total angular momentum (TAM) per photon is expressed as ±*ℏ* + *ℓℏ*^1^. Notably, vortex beams have been widely exploited in classical and quantum communications^9–12^, micromanipulation^13–15^, and other applications^16–22^, and have been studied beyond the domain of optics, for, e.g., acoustic and electron vortex beams^23,24^, to name a few.

Optical vortex beams are now generated by several techniques, including forked holograms^25^, spiral plates^26^, digital holography^27^, and spin–orbit-mediated metasurfaces^28,29^: q-plates^30^ and J-plates^31,32^. The common strategy is to create a phase singularity in the wavefront of incident light. Spin–orbit coupling, in particular, accomplishes this by imparting an azimuthally varying topological phase shift, known as the Pancharatnam–Berry (or geometric) phase^33,34^, which arises when the polarization state of incident light traverses a path on the Poincaré sphere. Using schemes with locally varying birefringence, these polarization transformations can be engineered, point-by-point, with subwavelength resolution, sculpting the incident wavefront into arbitrary vortex structures at-will^29,35,36^. In this process the photon’s SAM, associated with its polarization, is converted to OAM^37^; hence the term *spin–orbit coupling*. Notably, *q*-plates are among the earliest manifestations of this type of interaction albeit limited to converting circularly polarized light into a conjugate pair of OAM beams (with topological charges ±*ℓ*)^30^. This constraint was alleviated by J-plates which enable the conversion of any two arbitrarily chosen orthogonal polarization states to an arbitrary pair of OAM beams^31^—a versatility that was further enhanced using cascaded J-plates to decouple the input and output polarization states^32^. The majority of these phase plates, however, have been designed to generate OAM beams with a topological charge that remains fixed with propagation in free space, as required by OAM conservation^38,39^.

Digital holography has enabled more intriguing vortex beams in which the topological charge can spatially vary along the propagation direction^40–45^. The modulation in the values of *ℓ* and OAM is now well understood to occur only locally without disturbing the global angular momentum^46,47^. Besides OAM, much progress has been made to structure SAM—i.e., light’s polarization—along the optical path using spatial light modulators (SLMs)^48–51^, microchip lasers^52–54^, and metasurface optics^55,56^. Structured light of this nature offers extra degrees-of-freedom which can readily expand communication channel capacities^57^, suggest novel sensing schemes^56,58^, and enable versatile light–matter interaction in three dimensions. Existing techniques for generating these light structures, however, have primarily modulated either its spin^48–52,55,56^ or orbital components^40–45^. Simultaneous control of light’s TAM along the propagation direction is still at its infancy, mainly focusing on rotating the linear polarization state with limited control over its ellipticiy, and has heavily relied on SLMs which require multiple interactions with incident light, additional bulk optics, and precise alignment to achieve polarization control^41^, while only handling a fixed input polarization. On the other hand, microlaser chip configurations^52–54^ provide a more compact approach to this problem, but they rely on non-linear interactions and a gain medium which might not be a viable option in some applications. These limitations have been mitigated with metasurfaces, only partially, by modulating either polarization^55^ or OAM^43^ along the optical path. However, a device that can manipulate both components of angular momentum while handling incident light with any possible polarization has not been demonstrated despite the advances it could bring. For instance, controlling light’s TAM, longitudinally, provides versatile means for imaging, manipulating, and sorting colloids of different chirality, shapes, and sizes^14^ along the beam’s axis—remotely—with a single laser beam, and without additional bulk optics. It also suggests free-space communication scheme in which multiple receivers can detect different SAM–OAM combinations by probing the transmitted beam at multiple locations along its path, thus enhancing the channel capacity^57^. In this spirit, one can intercept the beam at different axial positions to potentially couple different OAM modes into different optical fibers in a multi-core array. Structured light of this kind can also be exploited in refractive index sensing whereby the OAM–SAM profile of the beam, detected at a fixed distance in an unknown fluid, will change its topology depending on the refractive index, enabling accurate detection of the unknown index over wide dynamic range^56,58^. The few attempts in this area have primarily exploited either OAM or polarization with no regard to TAM and have relied on bulky setups mediated by SLMs.

Here, we report on general spin–orbit interactions that address these gaps. We introduce a class of compact and versatile devices—dubbed TAM plates—which can operate on any incident polarization while controlling both the SAM and OAM of light along the propagation direction. We present a unified approach to realize these plates and demonstrate two broad classes. The first one consists of polarization-controlled devices which can couple any chosen pair of orthogonal polarization states into two distinct spatially variable vortex beams (i.e., OAM beams whose topological charge evolves from one state to another with propagation). Hence, the device can switch between two propagation-dependent vortex beams by changing the incident polarization; we refer to this device as polarization-switchable. The second, and more general, class of TAM plates are versatile; they are designed to operate on any incident polarization, enabling both the OAM and the polarization state to be structured along the optical path. Versatile spin–orbit coupling of this nature becomes particularly useful in micromanipulation where dynamic control over light’s angular momentum, across multiple planes, can be rapidly achieved by changing the incident polarization. To achieve these goals, we create superpositions of OAM modes with different wavenumbers, polarization, and OAM value. This produces an ensemble in which the OAM and SAM components beat along the propagation direction. We realize this profile via a single compact dielectric metasurfaces composed of shape-birefringent unit cells; i.e., rectangular nanofins whose retardance can be adjusted by varying their dimensions. Unlike previous OAM plates^30,31^, which predominantly lacked amplitude control, affecting the OAM mode purity^59,60^ and producing undesired hypergeometric modes^61^, our TAM plates can control the phase, amplitude, and polarization of incident light, simultaneously. To realize our designs, we use a recently developed matrix-based holography technique which enables complex modulation of light, on two orthogonal polarization channels, using a phase-only (lossless) platform^55^. Our work advances state-of-the-art techniques for vortex beam manipulation and extends the applications of structured light to dense data communication, light–matter interaction, and sensing^58^.

## Results

### Concept

**Fig. 1:**
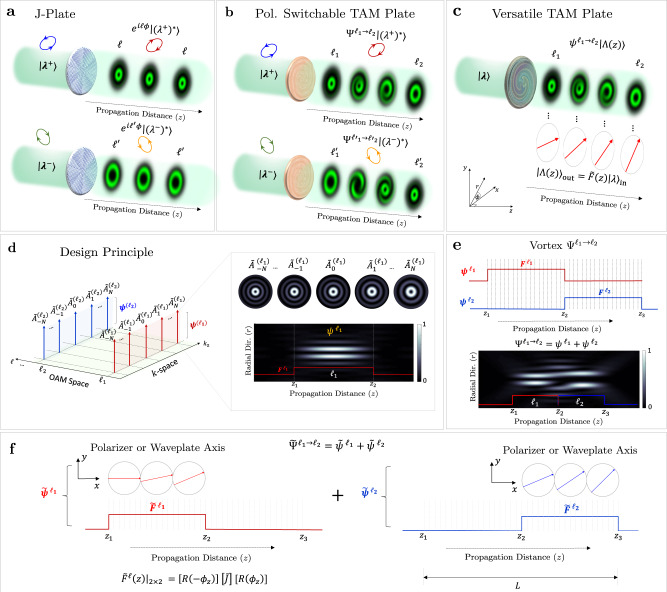
Concept of polarization-switchable and versatile TAM plates. **a** Conventional J-plates can impart two independent, propagation-invariant, azimuthaly varying phase profiles *e*^*iℓϕ*^ and ${e}^{i\ell ^{\prime} \phi }$ on the two eigen-polarization states, $\left|{\lambda }^{+}\right\rangle$ and $\left|{\lambda }^{-}\right\rangle$, while reversing their polarization handedness at the output. **b** A schematic of the proposed polarization-controlled TAM plate which can switch between two distinct spatially varying vortices, ${{{\Psi }}}^{{\ell }_{1}\to {\ell }_{2}}$ and ${{{\Psi }}}^{{\ell }_{1}^{\prime}\to {\ell }_{2}^{\prime}}$, in response to the input eigen polarizations $\left|{\lambda }^{+}\right\rangle$ and $\left|{\lambda }^{-}\right\rangle$. **c** A versatile TAM plate can control the polarization (Λ) and orbital angular momentum along the optical path. Light of any arbitrary polarization incident on the device will encounter variable polarization transformations as if interacting with different polarizing elements (as shown by the red arrows). **d** Design principle: the spatially varying vortex, ${{{\Psi }}}^{{\ell }_{1}\to {\ell }_{2}}$, is constructed from two series of OAM modes, denoted as *ψ*^*ℓ*^, each composed of 2*N* + 1 Bessel functions carrying the same *ℓ* value and equally separated in a comb-like arrangement in *k*_*z*_-space. The inset depicts five co-propagating OAM modes within ${\psi }^{{\ell }_{1}}$ and their envelope (longitudinal profile). By engineering the complex weights of these modes, the longitudinal intensity profile of the resulting envelope, ${\psi }^{{\ell }_{1}}$, can follow the user-defined function *F*^*ℓ*^ along the *z*-direction. **e** ${{{\Psi }}}^{{\ell }_{1}\to {\ell }_{2}}$ is realized by superimposing ${\psi }^{{\ell }_{1}}$ and ${\psi }^{{\ell }_{2}}$, whose respective profiles *F*^*ℓ*^ were judiciously engineered to cause destructive (constructive) interference at precise locations along *z*, leading to desired topological charge transition *ℓ*_1_ → *ℓ*_2_ with propagation. **f** The target propagation-dependent polarization response of the versatile TAM plate takes the form of a 2 × 2 Jones matrix, ${\tilde{F}}^{\ell }$. In general, this can be a polarizer or waveplate-like response. Here, we depict a response that mimics a polarizer or waveplate rotating its fast axis along the optical path.

Our aim is to devise polarization-switchable and versatile plates that can tailor TAM along the beam’s axis. Bound by angular momentum conservation, however, this sought after goal can only be realized locally as will be shown. Figure 1 illustrates two broad categories of TAM plates in comparison with conventional J-plates. While J-plates can impart two distinct azimuthally varying phase profiles (${e}^{i{\ell }\phi }$ or ${e}^{i{\ell }^{\prime}\phi }$) on any pair of orthogonal polarizations^31^, TAM plates create a complex-weighted superposition of OAM modes with different wavenumbers, polarizations, and topological charges, producing an envelope whose TAM changes locally along the propagation direction. The first implementation of TAM plates is shown in Fig. 1b. By changing the incident polarization, the device output can switch between two distinct propagation-dependent vortices (or a weighted average of the two). We refer to these two incident polarization states as the eigen polarizations of the device, $\left|{\lambda }^{+}\right\rangle$ and $\left|{\lambda }^{-}\right\rangle$. The second, and more general, example of TAM plates is shown in Fig. 1c in which the device not only transforms incident light into a spatially varying vortex beam but also modifies its polarization state, at-will, at each plane thereafter—as if encountering polarizing elements with different retardance and/or orientation (*ϕ*_*z*_). In the following, we begin by constructing a vortex beam with longitudinally varying charge (OAM), assuming a fixed input polarization, by revisiting the scalar formulation presented in ref. ^40^, then expand on it to realize our desired polarization-switchable and versatile behavior.

### Theoretical formulation—scalar case

An optical vortex with longitudinally varying charge, denoted as ${{{\Psi }}}^{{\ell }_{1}\to {\ell }_{2}}$, is constructed from a superposition of co-propagating OAM modes with different *ℓ* values and wavevectors *k*_*z*_, as shown in Fig. 1b. Here, ${{{\Psi }}}^{{\ell }_{1}\to {\ell }_{2}}={\sum }_{\ell }{\psi }^{\ell }$ and each series *ψ*^*ℓ*^ consists of *ℓ*-valued OAM modes that are equally spaced in a comb-like arrangement with a separation of 2*π*/*L* in *k*_*z*_-domain, where *L* is the longitudinal extent of the beam, as shown in Fig. 1d. Accordingly, the metasurface plate implements two or more OAM combs in spatial frequency domain. Their spatial beating yields an envelope in which the effective OAM value is modulated with propagation distance (Fig. 1e). Importantly, the envelope of *ψ*^*ℓ*^ is readily engineered, by selecting suitable complex-valued scalar coefficients for each OAM mode in the ensemble, to follow the arbitrarily chosen intensity profile given by *F*^*ℓ*^. Without loss of generality, we set our OAM modes to be quasi-non-diffracting beams with amplitude given by the higher-order Bessel distribution and azimuthal phase dependence ~*e*^*iℓϕ*^. This choice is particularly convenient since the Bessel beams are immune to diffraction over relatively long distances which serves our purpose^62^. The spatially modulated ensemble Ψ is thus expressed as^40^

1 $${{\Psi }}(\rho ,\phi ,z,t)=\mathop{\sum}\limits_{\ell }{\psi }^{\ell }={e}^{-i\omega t}\mathop{\sum }\limits_{\ell =-\infty }^{\infty }\mathop{\sum }\limits_{m=-N}^{N}{A}^{\ell ,m}{J}_{\ell }({k}_{\rho }^{\ell ,m}\rho ){e}^{i\ell \phi }{e}^{i{k}_{z}^{\ell ,m}z},$$

where ${k}_{\rho }^{\ell ,m}$ and ${k}_{z}^{\ell ,m}$ are the transverse and longitudinal wavevectors, respectively, such that: ${({k}_{\rho }^{\ell ,m})}^{2}+{({k}_{z}^{\ell ,m})}^{2}={(\omega /c)}^{2}$, and *A*^*ℓ*,*m*^ are complex-valued scalar coefficients for each Bessel function ${J}_{\ell }({k}_{\rho }^{\ell ,m}\rho )$ in the series and are evaluated from the Fourier-like relation

2 $${A}^{\ell ,m}=\frac{1}{L}\int\nolimits_{0}^{L}{F}^{\ell }(z){e}^{-i\frac{2\pi }{L}mz}\ {{{{{{{\rm{d}}}}}}}}z,$$

where *F*^*ℓ*^(*z*) denotes the desired longitudinal intensity profile of each OAM series *ψ*^*ℓ*^ in the sum. When evaluated at any point on the central ring, the Bessel superposition of Eq. (1) reduces to a 1D Fourier series along the *z*-axis. By the virtue of *F*^*ℓ*^(*z*), and their different values of *k*_*z*_, the co-propagating OAM modes within each *ψ*^*ℓ*^ will constructively interfere over a user-defined space region(s) while destructively interfering in all other regions along the optical path, as shown in the inset of Fig. 1d. It follows that, by combining multiple *k*_*z*_-combs with different *ℓ* values while properly designing their respective profile *F*^*ℓ*^(*z*), the ensemble Ψ can exhibit a modulation in its topological charge, locally, with propagation. Figure 1e illustrates this concept where two OAM series, ${\psi }^{{\ell }_{1}}$ and ${\psi }^{{\ell }_{2}}$, are cascaded along the propagation direction to construct the evolving vortex denoted as ${{{\Psi }}}^{{\ell }_{1}\to {\ell }_{2}}$; this is realized by *switching on* and *off* each *ψ*^*ℓ*^ contribution along the propagation path, via interference (and the proper choice of *F*^*ℓ*^), leading to a transition in the effective topological charge from *ℓ*_1_ to *ℓ*_2_. Importantly, the evolution of OAM and charge occurs locally by redistributing the beam’s energy and momentum across its outer rings, producing the desired profile only at the beam’s center while conserving the global OAM^46^.

### Multidimensional case

The above strategy, however, constructs the longitudinally evolving scalar vortex ${{{\Psi }}}^{{\ell }_{1}\to {\ell }_{2}}$ under the assumption of a fixed input polarization. To create a polarization-switchable plate, for instance, one would need each OAM series *ψ*^*ℓ*^ to exhibit a polarization-dependent response akin to, for e.g., a polarizer. In this way, the device produces the propagation-dependent vortex, preferentially, in response to a user-defined input polarization. A mathematically elegant way to express a polarizing element that operates on incident fully polarized light (Jones vectors) is the Jones matrix formalism^63^. Therefore, to realize a polarization-dependent response, the scalar vortex ${{{\Psi }}}^{{\ell }_{1}\to {\ell }_{2}}$ itself should acquire the functional form of a 2 × 2 Jones matrix. To achieve this, we revisit the expression of *ψ*^*ℓ*^ in Eq. (1) and let the scalar complex-valued coefficients *A*^*ℓ*,*m*^ of each Bessel function become Jones matrices instead, which we will denote as ${\tilde{A}}^{\ell ,m}$. Here, we adopt the notation (${\tilde{\;}}$) to express 2 × 2 Jones matrices. To obtain these matrix-valued coefficients^55,64^, we further modify Eq. (2) allowing it to acquire an arbitrary user-defined 2 × 2 Jones matrix profile ${\tilde{F}}^{\ell }$; this ${\tilde{F}}^{\ell }$ will dictate the target polarization response of each OAM series ${\tilde{\psi }}^{\ell }$ along the optical path. Consequently, Eqs. (1) and (2) will be expressed as

3 $$\tilde{{{\Psi }}}(\rho ,\phi ,z,t)=\mathop{\sum}\limits_{\ell }{\tilde{\psi }}^{\ell }={e}^{-i\omega t}\mathop{\sum}\limits_{\ell }\mathop{\sum }\limits_{m=-N}^{N}{\tilde{A}}^{\ell ,m}{J}_{\ell }({k}_{\rho }^{\ell ,m}\rho ){e}^{i\ell \phi }{e}^{i{k}_{z}^{\ell ,m}z},$$

and

4 $${\tilde{A}}^{\ell ,m}=\frac{1}{L}\int\nolimits_{0}^{L}{\tilde{F}}^{\ell }(z){e}^{-i\frac{2\pi }{L}mz}\ {{{{{{{\rm{d}}}}}}}}z.$$

In analogy with the scalar picture depicted in Fig. 1e, a matrix-valued superposition of two (or more) OAM waveforms ${\tilde{\psi }}^{\ell }$, now associated with judiciously designed polarization response ${\tilde{F}}^{\ell }$, can be cascaded along the optical path (Fig. 1f). Choosing the target response ${\tilde{F}}^{\ell }$ as a 2 × 2 Jones matrix of a polarizer will create a vortex ${\tilde{\psi }}^{{\ell }_{1}\to {\ell }_{2}}$, preferentially, in response to the eigen-polarization analyzed by ${\tilde{F}}^{\ell }$. Furthermore, a second matrix-valued vortex, ${\tilde{\psi }}^{{\ell }_{1}^{\prime}\to {\ell }_{2}^{\prime}}$, that responds to the other (orthogonal) polarization with a different set of *ℓ* values, can be combined on the same device. In this case, the superposition $\tilde{{{\Psi }}}={\tilde{{{\Psi }}}}^{{\ell }_{1}\to {\ell }_{2}}+{\tilde{{{\Psi }}}}^{{\ell }_{1}^{\prime}\to {\ell }_{2}^{\prime}}$ creates the vortices ${{{\Psi }}}^{{\ell }_{1}\to {\ell }_{2}}$, ${{{\Psi }}}^{{\ell }_{1}^{\prime}\to {\ell }_{2}^{\prime}}$, or a combination thereof, depending on the incident polarization—serving as a polarization-switchable TAM plate (Fig. 1b). In addition to this polarizer-like behavior, the target polarization response ${\tilde{F}}^{\ell }$ can be chosen as a variable waveplate which enacts propagation-dependent retardance on incident light^55^, thereby modifying its state of polarization (besides the OAM) along the direction of propagation, *z* (Fig. 1f). In this case, light interacting with the device will experience a different waveplate-like transformation at each plane thereafter leading to an evolution in its TAM (spin and orbital) along the optical path (Fig. 1c). Here, solving for $\tilde{{{\Psi }}}(\rho ,\phi ,z=0)$ defines the metasurface profile in real space. Light of any polarization incident on this device will be transformed to a diffraction-less vortex beam whose angular momentum evolves along *z*, in real space as well. This is fundamentally different from previous efforts in OAM metasurface holography which manipulate OAM superpositions in momentum space^65,66^. In the following, we start with a simple example that shows how this design strategy can realize a polarization-sensitive longitudinally evolving vortex then discuss its means of implementation using metasurface optics. Afterwards, we demonstrate the two broad classes of TAM plates, depicted in Fig. 1b, c, by allowing ${\tilde{F}}^{\ell }$ to take the form of either i) polarizers or ii) waveplates, respectively.

### Workflow: Polarization-sensitive vortex

We illustrate how the theory above can translate into a concrete workflow for designing a metasurface enacting a desired longitudinally varying OAM transformation. As an example, let us design a device creating spatially varying vortex ${\tilde{\psi }}^{\ell }$ that is sensitive to a particular incident polarization. To achieve this, we let the target polarization function ${\tilde{F}}^{\ell }$ take the functional form of a polarizer which can be represented by the 2 × 2 Jones matrix obtained from the outer product

5 $${\tilde{J}}_{{{\mbox{P}}}}=a\left|p\right\rangle \left\langle q\right|.$$

Here, $\left|q\right\rangle$ is the Jones vector being analyzed for; this is the incident polarization state for which the device will preferentially generate the desired vortex while extinguishing the orthogonal polarization, $\left|{q}^{\perp }\right\rangle$. $\left|p\right\rangle$, on the other hand, denotes the polarization state produced by the plate, and *a* is an arbitrary complex scalar-valued coefficient. For a conventional polarizer, the output polarization $\left|p\right\rangle$ is the same as the analyzed state, $\left|q\right\rangle$. As an example, we set this device to create a longitudinally evolving vortex with maximum intensity in response to incident *x*-polarization, $\left|q\right\rangle ={[1 \ \ \ 0]}^{{{\mbox{T}}}}$, while responding to other input polarizations with lower intensity in accordance to Malus law. The design flow is as follows: (i) The target polarization function ${\tilde{F}}^{\ell }$ is first specified over a finite distance, *L*. In this example, ${\tilde{F}}^{\ell }$ is obtained by substituting $\left|q\right\rangle$ and $\left|p\right\rangle$ in Eq. (5) such that ${\tilde{F}}^{\ell }$ takes the form of a linear polarizer. (ii) This ${\tilde{F}}^{\ell }$ is then substituted in Eq. (4) to obtain the matrix-valued coefficients ${\tilde{A}}^{\ell ,m}$. (iii) The coefficients ${\tilde{A}}^{\ell ,m}$ are used in Eq. (3) to evaluate ${\tilde{\psi }}^{\ell }(\rho ,\phi ,z=0)$, which represents a spatially varying distribution of 2 × 2 Jones matrices in the transverse (i.e., *x*–*y*) plane. This three-step process is repeated for different *ℓ*-valued OAM series ${\tilde{\psi }}^{\ell }(\rho ,\phi ,z=0)$ to design the full spin–orbit response of the device; i.e., endowing the output beam with all desired OAM values and polarization states over the predefined space region(s). (iv) These ${\tilde{\psi }}^{\ell }$ are added to obtain the superposition $\tilde{{{\Psi }}}={\sum }_{\ell }{\tilde{\psi }}^{\ell }(z=0)$ which defines the target phase-amplitude-polarization profile that we wish to implement on our metasurface—a distribution of Jones matrices that act, point-by-point, on incident Jones vectors to produce the intended vortices. Supplementary Figure 1 shows the response of a metasurface, based on this design principle, which generates a propagation-dependent vortex, preferentially, for input *x*-polarization. In the following, we show the implementation of these metasurface optics.

### Metasurface implementation

The two-dimensional (2D) profile of our devices is of the form $\tilde{{{\Psi }}}(z=0)$—a distribution of spatially varying 2 × 2 Jones matrices. Implementing a profile of this nature using conventional phase masks or SLMs, which all assume scalar-valued pixels, is cumbersome as it mandates multiple (possibly three or more) interactions with incident light^67^. To implement this matrix-valued profile one needs a scheme comprising locally varying birefringent pixels that operate on two orthogonal polarizations, simultaneously. To achieve this task, we deploy metasurfaces made of arrays of nano-structured birefringent unit cells^35,68^; linearly birefringent nanofins which can, point-by-point, be regarded as tunable waveplates. These nanofins can impart a phase shift between [0,2*π*] along their two anisotropy axes as detailed more fully in the “Methods” and Supplementary Note 1. Due to the lossless nature of these waveplate-like pixels, however, our metasurface does not modify the amplitude of incident light, only its phase. To overcome this obstacle, we adopt a recently developed matrix-based holographic technique which allows us to control not only the polarization and phase but also the amplitude across the output wavefront^55^. This method is inspired by the concept of dual phase holography^69,70^ which relies on engineering the phase-shift between two adjacent pixels on a hologram to realize complex (phase and amplitude) modulation of light in the far-field. Here, we consider the matrix-analog of this problem. Our approach, dual-matrix holography is summarized as follows: (i) The target metasurface profile $\tilde{{{\Psi }}}$ is first computed via Eq. (3), at *z* = 0, yielding a distribution of 2-by-2 matrices whose elements consists of mixed amplitude and phase. (ii) Each 2-by-2 matrix is decomposed into a sum of two phase-only (i.e., unitary) matrices. Here, phase-only matrices refer to matrices whose eigenvalues are strictly phase quantities. These unitary matrices are now compatible with our metasurface scheme; they can be realized by nanofins of different dimensions and orientations. (iii) At each location of the metasurface, only one of the two matrices is implement. (iv) By interlacing the two distributions of unitary matrices following a checkerboard pattern on the metasurface, while obeying Nyquist’s sampling criterion, the full complex spectrum (i.e., amplitude and phase) can be retrieved in the far-field (via a lens) given a band-limited waveform. (v) Lastly, through an inverse Fourier transform, implemented by a second lens, the target metasurface response can be constructed in real space. This differs from other approaches that rely on subwavelength interference to realize amplitude modulation via reflection^71^ or by varying the nanofin height to achieve complex modulation on one polarization channel^66^. The mathematical description of our method can be found in Supplementary Note 2.

### Experimental setup

**Fig. 2:**
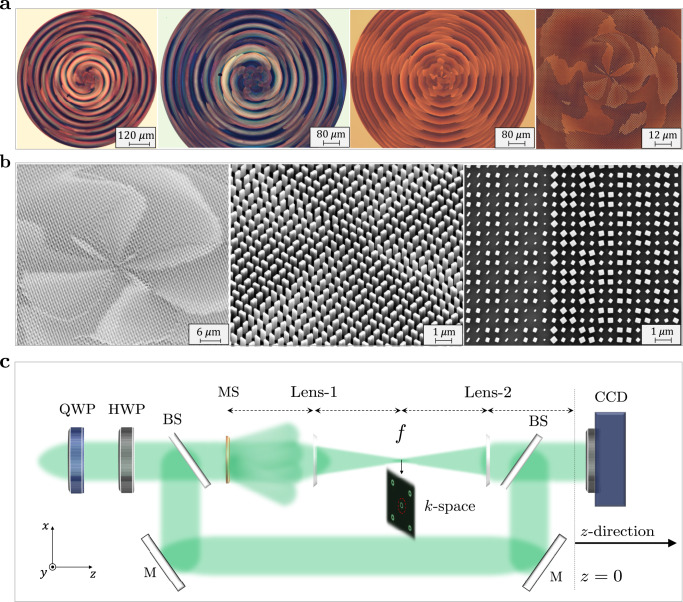
Fabricated devices and experimental setup. **a** Optical microscope images of sample fabricated TAM plate devices exhibiting a flower petal-like structure. **b** Scanning electron microscope (SEM) images verifying the smooth sidewall profile of the individual nanofins. The checkerboard-like pattern observed in the nanofin orientations signifies the underlying dual-matrix holography (DMH) implementation; each element of the target dual-matrix hologram is realized on the metasurface by four nanofins. **c** Experimental setup (top view) used to characterize the devices: a quarter-waveplate (QWP) and half-waveplate (HWP) control the polarization of the beam incident on the metasurface (MS). The output response is filtered and imaged using a 4-*f* system (*f* = 5 cm) onto a charge-coupled device (CCD) camera. An interferometric setup, comprising beam splitters (BS) and mirrors (M), is deployed to interfere each vortex beam with a tilted Gaussian beam to characterize its *z*-dependent topological charge value.

The shape-birefringent metasurface platform in combination with dual-matrix holography can implement the desired amplitude–phase–polarization profiles of Eq. (3), realizing both polarization-switchable and versatile devices for TAM control. All devices were designed at *λ* = 532 nm wavelength and with a device diameter of 924 μm. The choice of the design parameters and its implications are discussed more fully in Supplementary Note 3. Each design has been fabricated via electron beam lithography and atom layer deposition^72^, as outlined in the “Methods”. Sample micrographs and SEM images of our fabricated devices are shown in Fig. 2a, b, respectively, expressing very characteristic petal-like flower patterns hinting at the devices’ function. After all, these plates sculpt an incident plane wave into a twisted light structure with a stack of outer rings (layers), which carry the beam’s momentum, reminiscent of a twisted flower.

The output response of our devices has been characterized using the 4-*f* optical system depicted in Fig. 2c^55^. The role of this 4-*f* system is twofold: (a) to construct the complex spectrum of the beam at the Fourier plane (output focal plane of lens-1), where it is spatially filtered from higher diffraction orders in *k*-space and (b) to transform the filtered k-domain spectrum (circled in red) back to real space through an inverse Fourier transform via lens-2. The higher diffraction orders here act as energy loss channels that allow the lossless waveplate-like metasurface unit cells to achieve amplitude modulation without violating energy conservation. Although the nanofin separation is subwavelength, each four adjacent nanofins collectively modulate the amplitude response giving rise to these higher orders (Supplementary Note 2). The intensity of those orders relative to the signal is dictated by the incident polarization (Supplementary Note 4). The generated beam at the output of the 4-*f* system is then recorded by a CCD camera at multiple planes along the propagation direction (*z*) to reconstruct its longitudinal intensity profile, where the *z* = 0 plane lies at the output focal plane of lens-2. Additionally, an interferometric setup comprising beam splitters and mirrors was installed to interfere each vortex beam with a tilted reference Gaussian beam in order to identify its evolving (*z*-dependent) topological charge value. In the following, we demonstrate the two broad classes of our TAM plates.

### TAM Plate-1: Spin-controlled vortices with evolving OAM

To realize a polarization-switchable TAM plate, we implement two matrix-valued vortex profiles, ${\tilde{{{\Psi }}}}^{{\ell }_{1}\to {\ell }_{2}}$ and ${\tilde{{{\Psi }}}}^{{\ell }_{1}^{\prime}\to {\ell }_{2}^{\prime}}$, with a single metasurface while defining their target polarization response as analyzers for any orthogonal pair of user-defined states: $\left|{\lambda }^{+}\right\rangle$ and $\left|{\lambda }^{-}\right\rangle$, respectively. Notably, by superposing ${\tilde{{{\Psi }}}}^{{\ell }_{1}\to {\ell }_{2}}$, analyzing for $\left|{\lambda }^{+}\right\rangle$, and another, ${\tilde{{{\Psi }}}}^{{\ell }_{1}^{\prime}\to {\ell }_{2}^{\prime}}$, analyzing for $\left|{\lambda }^{-}\right\rangle$, we obtain a 2 × 2 spatially varying operator, $\tilde{{{\Psi }}}$, that enables a polarization-switchable OAM response. This TAM plate will produce an OAM beam changing its *ℓ* value, from *ℓ*_1_ to *ℓ*_2_, in response to the incident polarization $\left|{\lambda }^{+}\right\rangle$, while responding to the orthogonal polarization $\left|{\lambda }^{-}\right\rangle$ with a completely distinct OAM beam, evolving from ${\ell }_{1}^{\prime}$ to ${\ell }_{2}^{\prime}$. Importantly, the choice of these $\left|\lambda \right\rangle$ and *ℓ* values is completely arbitrary. Our design approach allows any two orthogonal polarizations to be chosen as the eigen polarizations of the device. To reconcile this recall that, in a linear polarization basis, any two orthogonal polarization states can be generally expressed as

6 $$\left|{\lambda }^{+}\right\rangle =\left[\begin{array}{l}\cos (\chi )\\ {e}^{i\delta }\sin (\chi )\end{array}\right],\left|{\lambda }^{-}\right\rangle =\left[\begin{array}{l}-\sin (\chi )\\ {e}^{i\delta }\cos (\chi )\end{array}\right].$$

Here, *δ* and *χ* set the ellipticity and orientation of the polarization state. Our aim is to perform the mapping $\left|{\lambda }^{+}\right\rangle \to {\tilde{{{\Psi }}}}^{{\ell }_{1}\to {\ell }_{2}}\left|{({\lambda }^{+})}^{* }\right\rangle$ and $\left|{\lambda }^{-}\right\rangle \to {\tilde{{{\Psi }}}}^{{\ell }_{1}^{\prime}\to {\ell }_{2}^{\prime}}\left|{({\lambda }^{-})}^{* }\right\rangle$. To demonstrate an instance of this spin–orbit mapping, we let our device implement the superposition $\tilde{{{\Psi }}}={\tilde{{{\Psi }}}}^{{\ell }_{1}\to {\ell }_{2}}+{\tilde{{{\Psi }}}}^{{\ell }_{1}^{\prime}\to {\ell }_{2}^{\prime}}$ such that ${\tilde{{{\Psi }}}}^{{\ell }_{1}\to {\ell }_{2}}={\tilde{\psi }}^{\ell = 1}+{\tilde{\psi }}^{\ell = -3}$ and ${\tilde{{{\Psi }}}}^{{\ell }_{1}^{\prime}\to {\ell }_{2}^{\prime}}={\tilde{\psi }}^{\ell = 2}+{\tilde{\psi }}^{\ell = -1}$. Hence, for a particular incident polarization, $\left|{\lambda }^{+}\right\rangle$, this TAM plate generates a spatially evolving vortex that changes its topological charge from *ℓ* = 1 to *ℓ* = −3 as it propagates, while producing a different vortex, evolving from *ℓ* = 2 to *ℓ* = −1, in response to the orthogonal input polarization, $\left|{\lambda }^{-}\right\rangle$. In our design, we set *χ* = *π*/4 and *δ* = −*π*/6 to define the two eigen polarizations of the device. This choice is completely arbitrary and shows the versatility of our design. Then, from Eq. (5), we obtained the two Jones matrices, analyzing for the chosen states $\left|{\lambda }^{+}\right\rangle$ and $\left|{\lambda }^{-}\right\rangle$. These Jones matrices represent the target polarization functions ${\tilde{F}}^{\ell }$ associated with ${\tilde{{{\Psi }}}}^{{\ell }_{1}\to {\ell }_{2}}$ and ${\tilde{{{\Psi }}}}^{{\ell }_{1}^{\prime}\to {\ell }_{2}^{\prime}}$, in Eqs. (3) and (4). We let ${\tilde{F}}^{\ell }$, associated with ${\tilde{\psi }}^{\ell = 1}$ and ${\tilde{\psi }}^{\ell = -3}$, take the form of a Jones matrix analyzing for the state $\left|{\lambda }^{+}\right\rangle$ over the space regions {8 mm ≤ *z* ≤ 16 mm} and {16 mm ≤ *z* ≤ 24 mm}, respectively, while setting ${\tilde{F}}^{\ell }$ associated with ${\tilde{\psi }}^{\ell = 2}$ and ${\tilde{\psi }}^{\ell = -1}$ as an analyzer for the orthogonal state $\left|{\lambda }^{-}\right\rangle$, over the same two regions. A device that implements this design, in essence, will produce Ψ^1→−3^ or Ψ^2→−1^ (or a combination thereof) depending on the incident polarization.

**Fig. 3:**
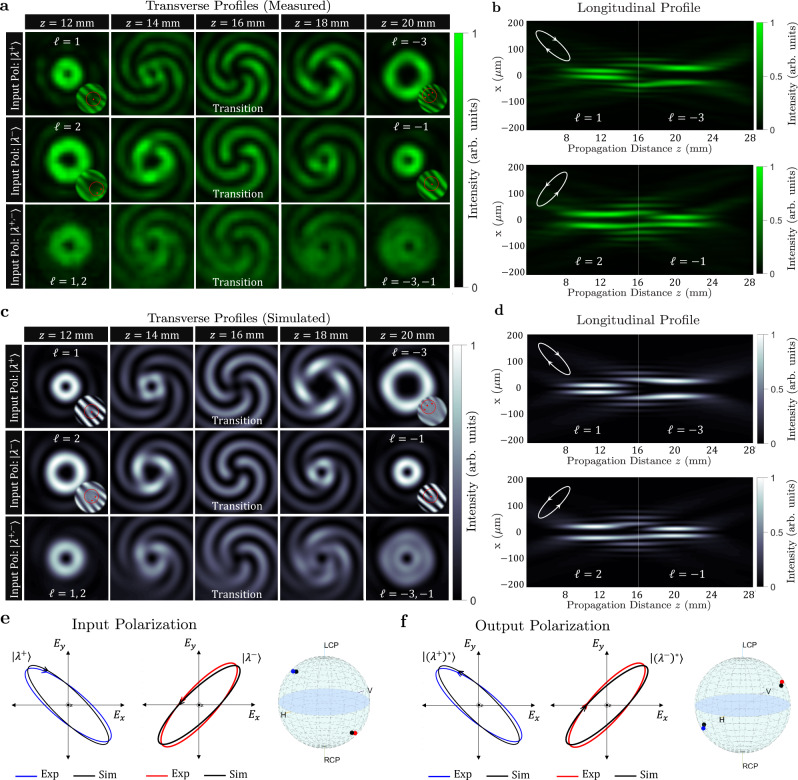
Polarization-switchable TAM plate with elliptical eigen polarizations. **a** Measured output transverse intensity profiles generated by the device in response to $\left|{\lambda }^{+}\right\rangle$ and $\left|{\lambda }^{-}\right\rangle$ polarizations, in addition to an equal weight of the two. The device responds to $\left|{\lambda }^{+}\right\rangle$ polarization by generating a vortex beam changing its charge from *ℓ* = 1 to *ℓ* = −3 as it propagates along the *z*-direction, whereas for the orthogonal polarization, $\left|{\lambda }^{-}\right\rangle$, a different vortex evolving from *ℓ* = 2 to *ℓ* = −1 is produced. The insets depict the topological phase dislocations obtained from an interferomteric measurement with a tilted Gaussian beam. At each *z*-plane, a mixture of the two OAM states can be generated by changing the weights assigned to the eigen polarizations incident on the device. **b** Measured longitudinal intensity profile at the output of the device under the two orthogonal incident polarizations, $\left|{\lambda }^{+}\right\rangle$ and $\left|{\lambda }^{-}\right\rangle$, confirming the generation of two distinct vortices with spatially evolving topological charges. **c**, **d** Simulated data corresponding to the measurements in **a**, **b**. **e**, **f** Measured and designed eigen polarizations at the input (**e**) and output (**f**) of the device. The chirality of incident light is inverted upon interacting with the device. Spatial dynamics of these beams can be found in Supplementary Movies 1 and 2.

Figure 3 reveals the consequence of this design strategy. The measured intensity profiles of the generated vortex beams are shown in Fig. 3a, b under the incident polarizations: $\left|{\lambda }^{+}\right\rangle$, $\left|{\lambda }^{-}\right\rangle$, and the polarization state in between. The device responds to $\left|{\lambda }^{+}\right\rangle$ polarization by generating an OAM beam evolving from *ℓ* = 1 to *ℓ* = −3—a behavior that is hinted at by observing the change in the ring size, further confirmed by the fork-like interference patterns, and in full agreement with the design. For the orthogonal incident polarization ($\left|{\lambda }^{-}\right\rangle$), however, the same device produces a completely distinct vortex exhibiting a topological transition from *ℓ* = 2 to *ℓ* = −1, as expected. Here, we chose positive and negative values for *ℓ* to demonstrate the versatility of our approach. Each topological transition is associated with an overlap region, *z* = 16 mm, where the vortex beam collapses to a spiraling pattern with ∣*ℓ*_2_ − *ℓ*_1_∣ interference fringes. When illuminated by other polarizations, the device generates a weighted superposition of the two OAM states, as shown in the same figure. The spatial evolution of these vortices can be viewed in Supplementary Movies 1 and 2. These measurements are in excellent agreement with the simulated data in (c). Additionally, the measured and simulated longitudinal profiles are shown in Fig. 3b, d, respectively, confirming the spatially varying spin–orbit coupling mediated by our device. In Supplementary Fig. 4, we examine the spin and OAM densities of these vortices and show that, despite their local variation, both quantities are globally conserved, separately.

We performed Stokes polarimetry^73^ to characterize the polarization behavior at the input and output of the device (see “Methods”). Figure 3e illustrates this characterization where the normalized electric field vector of the incident beam, $\left|{\lambda }^{+}\right\rangle$ and $\left|{\lambda }^{-}\right\rangle$, is plotted and displayed on the Poincaré sphere which visualizes all possible polarization states. The measured eigen polarizations of the device exhibit very good agreement with the designed states. Upon interacting with our TAM plate, these two orthogonal and elliptical states are mapped to the two distinct longitudinally varying vortices depicted in (a–d). Moreover, the input polarization states reverse their chirality at the output of the device, signified by a transition to the other hemisphere on the Poincaré sphere as shown in Fig. 3f. The reason for this flipping is explained in the “Methods” section. We emphasize that while we selected elliptical eigen-polarization states for this polarization-switchable TAM plate, our approach is general: the choice of $\left|{\lambda }^{+}\right\rangle$ and $\left|{\lambda }^{-}\right\rangle$ as well as their mapping to ${{{\Psi }}}^{{\ell }_{1}\to {\ell }_{2}}$ and ${{{\Psi }}}^{\ell_1 ^{\prime} \to \ell_2 ^{\prime} }$ at the output is completely specified by the design. To confirm this, we fabricated and tested another device with the two linear eigen polarizations, $\hat{x}$ and $\hat{y}$, performing the mapping $\hat{x}\to {{{\Psi }}}^{1\to 2}$ and $\hat{y}\to {{{\Psi }}}^{3\to -2}$, following the same design strategy above, the results of which agree with simulations very well as shown in Supplementary Fig. 5.

### TAM Plate-2: Versatile plates for structuring SAM and OAM

The second class of TAM plates are versatile devices that can operate on any incident polarization by shaping light into an optical vortex with longitudinally varying topological charge and polarization state. To achieve this behavior, we implement the matrix-valued vortex profile ${\tilde{{{\Psi }}}}^{{\ell }_{1}\to {\ell }_{2}}$ and set its target polarization response to be a waveplate with a propagation-dependent axis orientation. As an example, we define ${\tilde{{{\Psi }}}}^{{\ell }_{1}\to {\ell }_{2}}={\tilde{\psi }}^{\ell = 3}+{\tilde{\psi }}^{\ell = 2}$ and ${\tilde{F}}^{\ell }(z)$ as a 2 × 2 matrix of a quarter-waveplate whose angular orientation is a linear function of the propagation distance *z*, leading to a rotation in the fast axis from 0^∘^ to 90^∘^ over the range {10 mm ≤ *z* ≤ 22 mm}. Linearly polarized light incident on such a device will be sculpted into an optical vortex that changes its charge from *ℓ* = 3 to *ℓ* = 2 and its polarization will evolve from a linear to a circular state and vice versa (modifying its chirality and, hence, its SAM) as it propagates away from the device.

**Fig. 4:**
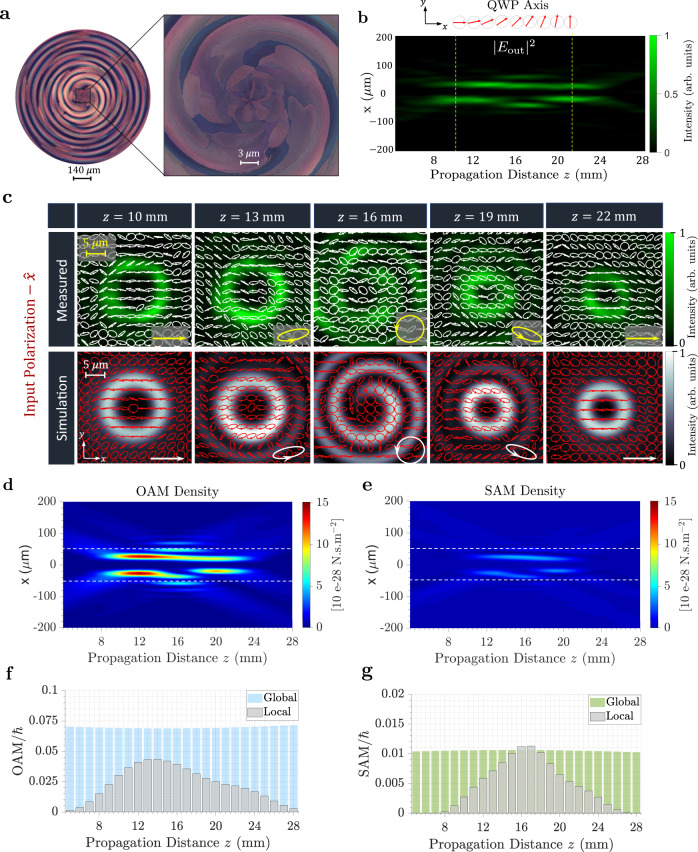
Versatile TAM plate for independent SAM and OAM control along the direction of propagation. **a** Optical microscope images of the device. **b** Measured longitudinal intensity profile at the output in response to incident *x*-polarization. The polarization response here is chosen to mimic a quarter-waveplate (QWP) with rotating fast axis, as shown by the red arrows. **c** Measured and simulated transverse intensity profiles of the generated vortex, Ψ^3→2^, in response to *x*-polarization, exhibiting a transition in the topological charge from *ℓ* = 3 to *ℓ* = 2. The state of polarization, obtained from Stokes polarimetry, is shown across the spatial extent of the beam, exhibiting a variation in the ellipticity at different propagation distances, as expected from a *z*-dependent quarter-waveplate-like device. **d**, **e** Simulated density maps depicting the OAM density (**d**) and SAM density (**e**) along the direction of propagation. **f** OAM values plotted as a function of propagation distance, obtained by integrating the OAM density in (**d**) at each transverse plane. The integration is performed twice considering two different aperture sizes: 100 μm (marked by the dashed lines in (**d**)) and 500 μm. We refer to these integrated values as local and global OAM, respectively. **g** Local and global SAM obtained by integrating the SAM densities in (**e**) using the same limits of integration as (**f**). In both cases, while the OAM/SAM can vary locally their global quantities are always conserved with propagation.

Figure 4a displays micrographs of the fabricated device (which is 924 μm in diameter). The measured longitudinal intensity profile of the output beam is shown in Fig. 4b in response to *x*-polarized light and shows a reduction in the diameter of the output beam along the direction of propagation. This is consistent with the evolution of the topological charge from *ℓ* = 3 to *ℓ* = 2. The red arrows shown on top of the plot refer to the angular orientation of the rotating fast axis of the quarter-waveplate-like response enacted by the device. Additionally, the transverse intensity profiles were recorded at different propagation distances as shown in Fig. 4c, confirming the spatial evolution in the topological charge and in full agreement with simulations. To characterize the polarization behavior, we performed Stokes polarimetry on the recorded beam at each *z*-plane (see “Methods”). This helps visualize the spatial polarization distribution, pixel-by-pixel, as the beam propagates. It is observed that, under *x*-polarized illumination, the state of polarization at the center of the output beam evolves from horizontal to elliptical then becomes right-handed circularly polarized (at *z* = 16 mm) before it gradually reduces its eccentricity, retaining its initial linear polarization. This is consistent with the designed quarter-wave-plate-like response set by ${\tilde{F}}^{\ell }(z)$.

To study the evolution of the TAM, we evaluated the spin and OAM densities of the output beam, separately, as shown in Fig. 4d, e following the approach outlined in ref. ^74^ and further detailed in Supplementary Note 5. Notably, the OAM density exhibits a reduction along the optical path in agreement with the transition of the topological charge value from *ℓ* = 3 to *ℓ* = 2, whereas the SAM density reaches its peak value at *z* = 16 mm in agreement with the underlying RCP polarization state. Integrating the OAM/SAM densities over the transverse plane of the beam yields the OAM/SAM value at each propagation distance. We performed this integration over an aperture size of 100 μm, concentrated around the beam’s center, and a larger aperture of 500 μm, enclosing the outer rings of the beam. We refer to these integrated quantities as local and global OAM/SAM, respectively. Figure 4f, g reveals that while both components of angular momentum (spin and orbital) can vary locally with propagation, these quantities must be globally conserved. The conservation mechanism here relies on a judicious exchange of energy and momentum between the beam’s central ring and its side lobes.

**Fig. 5:**
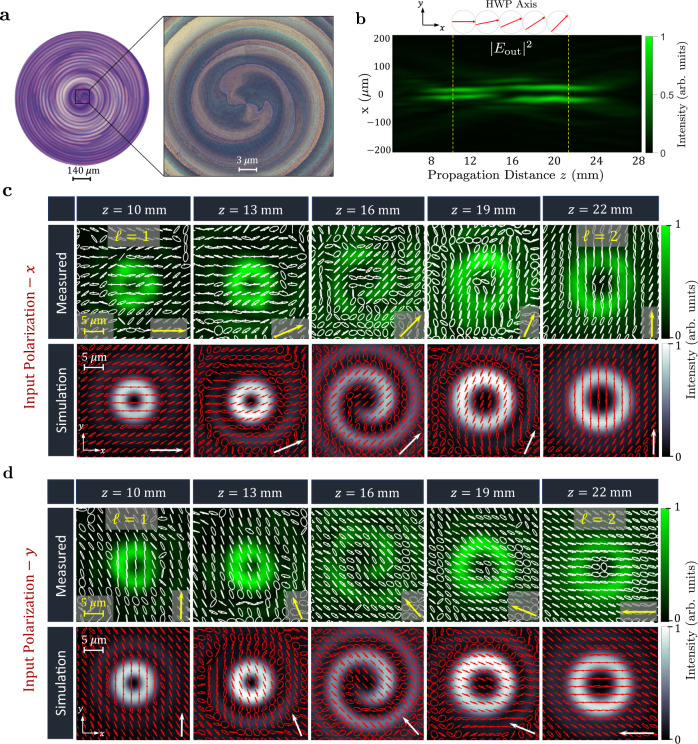
Versatile TAM plate for independent polarization and OAM control along the direction of propagation. **a** Optical microscope images of the device. **b** Measured longitudinal intensity profile at the output in response to incident *x*-polarization. The polarization response here is chosen to mimic a half-waveplate (HWP) with rotating fast axis, as shown by the red arrows. **c** Measured and simulated transverse intensity profiles of the generated vortex Ψ^1→2^ in response to *x*-polarization, exhibiting a transition in the topological charge from *ℓ* = 1 to *ℓ* = 2. The state of polarization, obtained from Stokes polarimetry, is shown across the spatial extent of the beam, exhibiting a rotation in the plane of linear polarization at different propagation distances. **d** Output response of the same device under *y*-polarization, depicting the evolution of the linear polarization from $\hat{y}$ to $\hat{x}$ along the direction of propagation and verifying that the TAM plates can impart its intended response on any incident polarization. The Stokes polarimetry and the evolution of the polarization state on the Poincaré sphere are depicted in Supplementary Fig. 6.

Lastly, to demonstrate the versatility of this design approach and its broad scope, we realized another TAM plate with a target polarization response that mimics a half-waveplate whose fast axis rotates with propagation. Figure 5a shows optical images of the fabricated sample with a diameter of 924 μm. The device is designed to create a vortex beam which changes its charge from *ℓ* = 1 to *ℓ* = 2 with propagation. The measured longitudinal intensity profile is depicted in Fig. 5b. The polarization response mimics a HWP retarder with a fast axis that rotates as denotes by the red arrows. When illuminated by *x*-polarized light such a device will adiabatically rotate the incident polarization by an amount of 90^∘^ as a function of propagation distance, as shown in the measured and simulated results of Fig. 5c. Importantly, these versatile TAM devices can operate on light of any incident polarization, exhibiting the designed transformation of OAM and polarization in response to all input polarizations. For example, when *y*-polarized light is incident on the same device it still rotates its polarization, gradually evolving to *x*-polarization with propagation, as shown in Fig. 5d. The evolution of the polarization state in polarization space, the Poincaré sphere, is shown in Supplementary Fig. 6.

## Discussion

In this work we presented a generalized approach, based on fully-analytical formulation, to design and implement a class of OAM plates—TAM plates—that can manipulate the TAM of incident light along the propagation direction, without setting constraints on the incident polarization. We demonstrated two classes of these devices; one that couples any pair of orthogonal polarizations, incident on the device, into two independent sets of vortex beams whose topological charge (wavefront’s helicity) can vary locally along the optical path, and another class of devices that can operate on any incident polarization by structuring its spin and orbital angular momenta, independently, along the optical path. Previous work of this nature either (i) demonstrated spin–orbit coupling into OAM modes with a fixed topological charge^30,31^ or (ii) considered the scalar treatment of the current problem, where a phase mask comprised of scalar pixels (realized by digital holography) sculpt an incident plane wave *with a fixed polarization*, after multiple interactions, into a spatially varying vortex beam^40–45^. To bridge this gap, we relied on Jones matrix formalism which allows a multifunctional response while considering all possible input polarizations, at once. Our devices can encode complex (phase-amplitude) vortex beams onto any two orthogonal polarization channels which can be either linear, elliptical, or circular, without imposing any constraints on the output OAM values.

Our work points towards more complex spin–orbit-mediated structured light generation with a compact and multifunctional device. It expands on previous efforts that exploited spin–orbit coupling for generating phase-only (~e^*iℓϕ*^) OAM modes by allowing more sophisticated transitions. Given our choice of Bessel functions as the OAM modes, our devices generate vortex beams characterized by a non-diffracting and self-healing behavior^62^ which becomes particularly desirable in micromanipulation and free-space optical communications. Notably, while we performed our experiments in the visible (532 nm), our design strategy is general and applies to other spectral regions. Our devices can also be scaled to generate optical vortices over a longer or shorter propagation range and can change the topological charge value at the output beyond two OAM modes, if desired, by changing the aperture size of the devices and the cone angles of the OAM modes following the systematic design considerations of axicons^62^, and as outlined in Supplementary Note 3. Furthermore, longitudinally varying OAM modes with very large differential topological charge number (Δ*ℓ* ~ 100) can be realized with our method as illustrated in Supplementary Fig. 7. This requires a metasurface with large diameter to ensure that the vortex beam is generated over a long propagation distance while overcoming diffraction effects (Supplementary Note 3).

Generating polarization-controlled structured light both in the transverse plane and along the propagation direction, as demonstrated in this work, can inspire alternative directions in science and technology. It advances the field of singular optics by enabling topologically complex states of light which in turn can lead to many interesting phenomena in quantum and classical entanglement and state generation^75–78^. Besides their potential application in light–matter interaction and free-space communications, the compact form of our devices enables their integration in laser cavities to generate topologically complex combinations of SAM and OAM states of light at the source^60,79,80^. Lastly, the multidisciplinary nature of angular momentum and singularity engineering across different fields may inspire related research efforts in the areas of microfluidics, acoustics, and electron beams, to name a few. We thus envision this work to enrich the science and applications of structured light and beyond.

## Methods

### Metasurface design

The 2D profile of our TAM plates is of the form ${\tilde{{{\Psi }}}}^{\ell }(z=0)$—a distribution of spatially varying 2-by-2 Jones matrices. An ideal candidate for implementing this matrix-valued profile is a metasurface—nano-structured 2D arrays of birefringent unit cells^35,68^. Here, we consider metasurfaces consisting of linearly birefringent waveplate-like elements which can, point-by-point, be expressed in the canonical form

7 $$\tilde{J}(x,y)={{{{{{{\bf{R}}}}}}}}(-\phi (x,y))\left[\begin{array}{ll}{e}^{i{\theta }_{x}(x,y)}&0\\ 0&{e}^{i{\theta }_{y}(x,y)}\end{array}\right]{{{{{{{\bf{R}}}}}}}}(\phi (x,y)).$$

The unit cells described by Eq. (7) take the form of rectangular dielectric nanofins with high refractive index contrast, serving as nano waveguides that support two orthogonal propagating modes, with different phase delays *θ*_*x*_ and *θ*_*y*_, due to anisotropy. Further, these nanofins can be rotated about the longitudinal axis by an angle *ϕ*, modifying their anisotropy axis in the *x*–*y* plane, as described by the rotation matrix **R**(*ϕ*). This design choice is very advantageous; it allows the implementation of a wide range of waveplate-like functions with continuously tunable values of *θ*_*x*_, *θ*_*y*_, and *ϕ*, at each point (*x*,*y*), by varying the nanofin dimensions and angular orientation. From Eq. (7), however, the middle matrix is diagonal and only has phase-only entries thus the resulting Jones matrix is both symmetric and unitary. These constraints set a limit on the Jones matrices that can be implemented by our metasurface: unitarity prohibits the realization of an absorptive operator, like, for e.g., a linear polarizer. Additionally, matrix symmetry, which stems from the linear form birefringence of the rectangular nanofins, implies that if a polarizer-like function is implemented then the output polarization is bound to be the complex conjugate of the polarization being analyzed for; enforcing the condition $\left|p\right\rangle ={\left|q\right\rangle }^{* }$ in Eq. (5)^64^. In this work, we have addressed the unitarity constraint with dual-matrix holography which allows us to encode phase-amplitude-polarization response using lossless waveplate-like unit cells (as further illustrated in Supplementary Note 2). However, we accept the limitation of matrix symmetry here and, hence, the chirality of incident light will always be reversed at the output.

### Simulation and modeling

The target phase-amplitude-polarization profile of each TAM plate was obtained by constructing the matrix-valued series $\tilde{{{\Psi }}}=\sum {\tilde{\psi }}^{\ell }(z=0)$ with the aid of Eq. (3). $\tilde{{{\Psi }}}$ represents a spatially varying distribution of 2-by-2 Jones matrices at each location on the metasurface. A MATLAB script was written to evaluate the spatially varying profile $\tilde{{{\Psi }}}$. Afterwards, Kirchhoff’s diffraction calculations were performed to set the aperture size that ensures efficient beam propagation over the desired longitudinal extent; i.e., a larger aperture enables longer propagation range following the same design considerations of an axicon. Note that an infinitely large aperture would generate a waveform that is periodic along the propagation direction, *z*. Once the proper aperture is specified, the target profile is then converted to a distribution of 2-by-2 unitary matrices to be compatible with the waveplate-like unit cells comprising our metasurface. A recently developed matrix-based holography technique, which we dub dual-matrix holography, was coded in a MATLAB script to perform this conversion. To realize our designs experimentally, a library of shape-birefringent nanofins (of different sizes and angular orientations) was generated by simulating the output phase and transmission response of each nanofin using a commercial FDTD solver. Finally, at each location of the metasurface, the nanofin that provides the closest realization of our target 2-by-2 Jones matrix was selected, following the selection rule in ref. ^81^. This process was repeated across the entire metasurface area to reach a CAD design for the final device.

### Device fabrication

A positive tone electron beam resist was spin coated on top of a fused silica substrate, ultimately defining the nanofins height. The resist was first baked then exposed using electron beam lithography (with accelerating voltage of 125 kV), writing the desired nanofin pattern. The exposed pattern was developed by submerging the sample in *o*-xylene for 60 s, creating the desired geometry of the individual nanofins. Afterwards, atomic layer deposition process was used to deposit TiO_2_, conformally filling the developed pattern. The excess TiO_2_ layer covering the device was etched away to the original height of the resist via reactive ion etching. Finally, the resist was chemically removed leaving the TiO_2_ nanofins surrounded by air.

### Polarization characterization

We performed Stokes polarimetry to measure the polarization state at the input and output of the device, thus retrieving the four-component polarization Stokes vector, $\overrightarrow{S}={({S}_{0},{S}_{1},{S}_{2},{S}_{3})}^{{{{{{{{\bf{T}}}}}}}}}$. Here, $\overrightarrow{S}$ quantifies the shape and orientation of the polarization ellipse at each point in space addition to the beam’s intensity and degree of polarization^73^. The Stokes parameters were obtained by rotating a polarizer (Pol) and a quarter-waveplate (QWP) before the CCD to analyze for the polarization states: 0^∘^, 45^∘^, 90^∘^, and right-hand circular polarization (RCP). We denote the corresponding intensities as ${I}_{{0}^{\circ }},{I}_{4{5}^{\circ }},{I}_{9{0}^{\circ }},$ and *I*_RCP_, respectively. At each *z*-plane, the four Stokes parameters were obtained as follows: ${S}_{0}={I}_{{0}^{\circ }}+{I}_{9{0}^{\circ }}$, ${S}_{1}={I}_{{0}^{\circ }}-{I}_{9{0}^{\circ }}$, ${S}_{2}=2({I}_{4{5}^{\circ }})-({I}_{{0}^{\circ }}+{I}_{9{0}^{\circ }})$, and ${S}_{3}=2({I}_{{{\mbox{RCP}}}})-({I}_{{0}^{\circ }}+{I}_{9{0}^{\circ }})$.

## Supplementary information

Supplementary Information

Description of Additional Supplementary Files

Supplementary Movie 1

Supplementary Movie 2

Supplementary Movie 3

Supplementary Movie 4

## Data Availability

All key data generated and analyzed are included in this paper and its supplementary information. Additional data sets that support the plots within this paper and other findings of this study are available from the corresponding author upon reasonable request.

## Code availability

The codes and simulation files that support the plots and data analysis within this paper are available from the corresponding author upon reasonable request.
