# Supplementary material for: Structuring total angular momentum of light along the propagation direction with polarization-controlled meta-optics
Source: Nat Commun. 2021 Oct 29;12:6249. doi: 10.1038/s41467-021-26253-4 (PMC8556329; doi:10.1038/s41467-021-26253-4)
Supplement: Supplementary file 1 — Supplementary Information [file 41467_2021_26253_MOESM1_ESM.pdf]

Supplementary Information
Structuring total angular momentum of light along the propagation direction with polarization-controlled meta-optics

Ahmed H. Dorrah[1,*], Noah A. Rubin[1], Michele Tamagnone[1,2], Aun Zaidi[1] & Federico Capasso[1,*]

[1] *Harvard John A. Paulson School of Engineering and Applied Sciences, Harvard University, Cambridge, Massachusetts 02138, USA*
[2] *Fondazione Istituto Italiano di Tecnologia, Genova, Italy.*
[*] *dorrah@seas.harvard.edu; capasso@seas.harvard.edu*

Contents

Supplementary Note 1: Metasurface Nanofin Response **2**

Supplementary Note 2: Dual Matrix Holography **3**

Supplementary Note 3: Design Considerations and Beam Dimensions **5**

Supplementary Note 4: Polarization-Sensitive Vortex **6**

Supplementary Note 5: Angular Momentum Density Calculation **6**

Supplementary Figure 1 **9**

Supplementary Figure 2 **10**

Supplementary Figure 3 **11**

Supplementary Figure 4 **12**

Supplementary Figure 5 **13**

Supplementary Figure 6 **14**

Supplementary Figure 7 **15**

Supplementary References **16**

Supplementary Note 1: Metasurface Nanofin Response

TAM plates were realized using nano-structured metasurfaces with rectangular dielectric nanofins of high index contrast. The nanofins were made of Titanium Dioxide (TiO_2) with a fixed height of 600 nm on top of a glass substrate, as depicted in Supplementary Fig. 1(a). These unit cells support two propagating modes which due to anisotropy experience different indices (phase delays). Each nanofin can be regarded as a waveplate-like element given by the 2-by-2 Jones matrix

$$\tilde{J}(x,y) = \mathbf{R}(-\phi(x,y)) \begin{bmatrix} e^{i\theta_x(x,y)} & 0 \\ 0 & e^{i\theta_y(x,y)} \end{bmatrix} \mathbf{R}(\phi(x,y)). \tag{1}$$

Notably, the phase retardances (θ_x and θ_y) in Eq. (1) are readily tuned by varying the dimensions of the nanofins in the transverse (i.e., x-y) plane, whereas the rotational angle ϕ can be adjusted by changing the nanofin's angular orientation about the longitudinal axis, z.

Supplementary Figs. 1(b) and (d) show the phase shift and transmission response imparted by each nanofin on an x-polarized incident plane wave, as a function of the nanofin dimensions, obtained numerically via FDTD simulations. The phase shift was obtained by probing the center of a monitor located above the structure in the far field, whereas the power transmission here is defined as the total power passing through a monitor above the structure relative to the input power. The average transmission efficiency is $\sim 70\%$. Supplementary Fig. 1(c) depicts the complex transmission coefficient imparted on x- polarized light, $t = t_x e^{i\theta_x}$. Each dot corresponds to one simulated geometry. The radius from the origin corresponds to t_x and the angle of the dot relative to the x axis is θ_x. This plot suggests that our metasurface library can provide almost full $[0, 2\pi]$ phase coverage with quasi uniform transmission over large number of geometries. A similar response applies to y-polarized light, from symmetry. Each nanofin can thus impart two independent phase profiles on light polarized along its major and minor axes. Furthermore, the rotational degree-of-freedom of each nanofin, combined with its linear form birefringence, can be exploited to manipulate the incident polarization, point-by-point, as described by Eq. (1).

To avoid operation near resonances, which are typically associated with narrow bandwidth and low fabrication tolerance, we pick nanofins that maintain a uniform transmission; i.e., nanofins which modify the phase (retardance) of incident light without modulating its amplitude. Because of this choice, our metasurface scheme can be regarded as unitary or lossless. In the following section, we show how amplitude modulation can be realized in the far field by judiciously arranging each two adjacent phase-only nanofins.

Supplementary Note 2: Dual Matrix Holography

Our metasurface should ideally realize the target distribution $\tilde{\Psi}$, based on Eq. (3) in the main text. As discussed in Supplementary Note 1, however, our metasurface can introduce retardance with no amplitude modulation. To implement the desired distribution $\tilde{\Psi}$—whose eigenvalues can be a mix of amplitude and/or phase—on a unitary metasurface, we deploy a matrix-based holographic technique dubbed dual matrix holography [1]. Our approach is to, point-by-point, express an arbitrary matrix (with complex eigenvalues) as a sum of two unitary matrices whose eigenvalues are phase-only quantities. This is analogous to adding two phasors (vectors) with the same amplitude but different directions to modulate the amplitude of the resultant vector. If each of the two phasors covers a phase range of $[0, 2\pi]$, tracing a unit circle, then the amplitude of the resultant vector can be fully modulated; it can lie within that unit circle. Similarly, since our unitary matrices can enact a retardance between $[0, 2\pi]$ (see Supplementary Note 1) then the addition of two unitary matrices should, in principle, realize a non-unitary matrix in which the eigenvalues can be fully modulated in amplitude. To perform the matrix-analogue of this decomposition, we run the procedure below:

(a) The target matrix distribution $\tilde{\Psi}$ is normalized, locally, by dividing by the global maximum eigenvalue: $\tilde{\Psi}_{\text{norm}} = \frac{\tilde{\Psi}}{\max\{\text{eig}(\tilde{\Psi})\}}$, everywhere. This normalization means that our passive metasurface may only introduce a loss-like effect with no gain.

(b) Each matrix in $\tilde{\Psi}_{\text{norm}}$ is factorized via singular value decomposition (SVD) as $\tilde{\Psi}_{\text{norm}} = [WDV^T]$, where D is a 2-by-2 diagonal matrix with non-negative real singular values.

(c) The singular values of $\tilde{\Psi}_{\text{norm}}$, D_{11} and D_{22}, at each location are decomposed to a sum of two complex values $C_{ii}^{(1)} = (D_{ii} + i\sqrt{1 - D_{ii}^2})/2$ and $C_{ii}^{(2)} = (D_{ii} - i\sqrt{1 - D_{ii}^2})/2$, where i denotes the index of the diagonal entry (ii = 11 or 22)[1].

(d) The complex values $C_{ii}^{(1,2)}$ then become the new diagonal entries of two unitary matrices, with the same rotation matrices W and V^T, such that

$$\tilde{\Psi}_{\text{norm}}(r,\phi) = \tilde{\Psi}_{\text{norm},1} + \tilde{\Psi}_{\text{norm},2} = W \begin{bmatrix} C_{11}^{(1)}(r,\phi) & 0 \\ 0 & C_{22}^{(1)}(r,\phi) \end{bmatrix} V^T + W \begin{bmatrix} C_{11}^{(2)}(r,\phi) & 0 \\ 0 & C_{22}^{(2)}(r,\phi) \end{bmatrix} V^T. \tag{2}$$

By construction, $D_{ii} = C_{ii}^{(1)} + C_{ii}^{(2)}$ and $|C_{ii}^{(1,2)}| = 1$, $\forall r, \phi$. As such, $\tilde{\Psi}_{\text{norm}}$ is now decomposed into two unitary matrices, denoted as $\tilde{\Psi}_{\text{norm},1}$ and $\tilde{\Psi}_{\text{norm},2}$, where each is now in the compatible form to be implemented using our unitary dielectric metasurface comprised of waveplate-like unit cells. At each location of the metasurface, only one of the two unitary matrices is implemented. Through judicious interlacing of these two matrix distributions on a single metasurface, the intended phase-amplitude spectrum can be constructed in the far-field. To achieve this, $\tilde{\Psi}_{\text{norm},1}$ and $\tilde{\Psi}_{\text{norm},2}$ are periodically sampled by means of two complementary checkerboard patterns, M_1 and

[1]Here, the spatial dependence has been omitted for clarity

M_2, expressed as

$$M_{1,2}(r,\phi) = \frac{1}{2}\sum_{n=-\infty}^{\infty}\sum_{l=-\infty}^{\infty} I[\Lambda_{1,2}(n,l)]e^{i\frac{2\pi r}{p}(n\cos\phi + l\sin\phi)}, \tag{3}$$

$$\Lambda_{1,2}(n,l) = \cos\left[\frac{\pi(n\pm l)}{2}\right]\text{sinc}(\frac{n\pi}{2})\text{sinc}(\frac{l\pi}{2}). \tag{4}$$

Here, p defines the periodicity of the checkerboard pattern. In essence, Eq. (3) renders two complementary checkerboard patterns whose transverse profile at each location alternates between the 2-by-2 identity matrix I and the zero matrix. Multiplying $\tilde{\Psi}_{\text{norm},1,2}$ by $M_{1,2}$, locally, and adding is equivalent to interlacing $\tilde{\Psi}_{\text{norm},1}$ and $\tilde{\Psi}_{\text{norm},2}$ onto the same surface with periodicity p. Notably, allowing the periodicity p to fulfil the Nyquist limit enables the full reconstruction of the transfer function of $\tilde{\Psi}_{\text{norm}}(r,\phi,z=0)$ in the far-field [2]. In our design, we set $p=2$ so that each checkboard unit cell is composed of 4 nanofins. The interlaced pattern on the metasurface plane is given by

$$\tilde{\mathcal{I}}(r,\phi) = M_1(r,\phi)\tilde{\Psi}_{\text{norm},1}(r,\phi) + M_2(r,\phi)\tilde{\Psi}_{\text{norm},2}(r,\phi). \tag{5}$$

Equation (5) defines the target profile that we wish to implement at the metasurface plane. Each location of $\tilde{\mathcal{I}}(r,\phi)$ represents a 2×2 matrix that can be realized with a waveplate-like nanofin of the functional form of Eq. (1). The three-step selection process of each individual nanofin of the designed metasurface is summarized as follows [3]: a) The complex-valued errors ϵ_x and ϵ_y are first evaluated from $\epsilon_x = |t_{\text{avg}}e^{i\theta_{x,\text{target}}} - t_{\text{simulated}}e^{i\theta_{x,\text{simulated}}}|$ and $\epsilon_y = |t_{\text{avg}}e^{i\theta_{y,\text{target}}} - t_{\text{simulated}}e^{i\theta_{y,\text{simulated}}}|$, for all possible nanofin configurations, where t_{avg} is the mean transmission (averaged over all simulated nanofins), whereas $e^{i\theta}$ and t are the phase and power transmission of each nanofin. b) For each nanofin geometry, the maximum error $\epsilon_{\text{max}} = \max(\epsilon_x, \epsilon_y)$ is determined, and finally c) the configuration that minimizes ϵ_{max} is selected, yielding the final metasurface design. By taking the Fourier transform of $\tilde{\mathcal{I}}(r,\phi)$, it can be shown that $\mathcal{F}\{\tilde{\mathcal{I}}(r,\phi)\} = \tilde{\mathbf{H}}_1(u,v) + \tilde{\mathbf{H}}_2(u,v)$, where

$$\tilde{\mathbf{H}}_1(u,v) = \sum_{n=-\infty}^{\infty}\sum_{l=-\infty}^{\infty}\Lambda_1(n,l)\tilde{\mathbf{\Pi}}(u-\frac{n}{p}, v-\frac{l}{p}), \tag{6}$$

and,

$$\tilde{\mathbf{H}}_2(u,v) = \sum_{n=-\infty}^{\infty}\sum_{l=-\infty}^{\infty}\Lambda_2(n,l)\tilde{\mathbf{\Omega}}(u-\frac{n}{p}, v-\frac{l}{p}). \tag{7}$$

Here, $\tilde{\Pi} = \mathcal{F}\{\tilde{\Psi}_{\text{norm},1}(r,\phi)\}$ and $\tilde{\mathbf{\Omega}} = \mathcal{F}\{\tilde{\Psi}_{\text{norm},2}(r,\phi)\}$. In essence, $\mathcal{F}\{\tilde{\mathcal{I}}(r,\phi)\}$ yields multiple copies of the spectra $\tilde{\mathbf{\Pi}}$ and $\tilde{\mathbf{\Omega}}$, about the points $(\frac{n}{p},\frac{l}{p})$. Since $\tilde{\Psi}(r,\phi,z=0)$ is band-limited, then its correct complex spectrum $[\tilde{\mathbf{\Pi}}(u,v)+\tilde{\mathbf{\Omega}}(u,v)]$ can be fully reconstructed by filtering in k-space provided that the sampling period p satisfies the Nyquist criteria (i.e., $\frac{1}{p} \geq \frac{\max(k_r^{(m)})}{2}$). Since $\tilde{\Psi}_{\text{norm}}(r,\phi,z=0) = \tilde{\Psi}_{\text{norm},1}(r,\phi)+\tilde{\Psi}_{\text{norm},2}(r,\phi)$, it follows that $\mathcal{F}\{\tilde{\Psi}_{\text{norm}}(r,\phi,z=0)\} = \tilde{\mathbf{\Pi}}(u,v)+\tilde{\mathbf{\Omega}}(u,v)$ and the desired spectrum is fully reconstructed. The desired distribution $\tilde{\Psi}(r,\phi,z=0)$, which defines the metasurface response, can then be retrieved in real space by performing an inverse Fourier operation.

Supplementary Note 3: Design Considerations and Beam Dimensions

In our designs, we set N=6 which yields 13 Bessel modes for each OAM series ψ^ℓ, as defined in Eq. (3) of the main text. Further, the longitudinal wavevectors $k_z^{(0)}$ were centered at a value of 0.999925 ω/c with a separation of $2\pi/L$ in k_z-space, where $L = 50$ mm. This choice of N and the wave vectors ensures operation in the paraxial regime where the scalar formulation given in Eq. (3) in the main text holds and the contribution of the longitudinal field component can be neglected. This choice of parameters is also intricately related to the generated beam's range and aperture size (diameter) of the plates. Essentially, a larger plate can generate vortex modes over a longer range—a relation that is precisely governed by the geometric argument of axicons [4]. After all, these TAM plates create non-diffracting Bessel-like vortex beams that can be visualized in terms of axicons of different cone angles. Note that a larger aperture size would generate multiple copies of the output waveform along the propagation direction due to the Fourier-like series underlying our Bessel beam superposition. In essence the equal separation between our OAM modes in k_z-space would create a waveform which is periodic along z, provided that the aperture is infinitely large. To this end, all devices were designed with a diameter of 924 μm; this aperture size is sufficient to ensure output beam propagation over the desired range, L, which has been confirmed by Kirchhoff's diffraction simulations [5]. In general, the minimum aperture diameter needed to generate a non-diffracting vortex over a range L is given by

$$D \geq 2L\sqrt{\left(\frac{k_0}{k_z^{m=-N}}\right)^2 - 1}, \tag{8}$$

where $k_0 = \omega_0/c$, and $k_z^{m=-N}$ is the smallest longitudinal wavenumber. This can be derived from the geometric argument of axicons in which k_z and k_ρ lie on the sides of a right angle triangle (i.e., along the z and the radial directions, respectively) such that $\frac{(D/2)}{L} = \frac{k_\rho}{k_z}$, as depicted in Supplementary Fig. 3. Equation (8) is valid for Bessel beams of 0-th order in which the energy is localized over a central spot. For $\ell \geq 1$ where the energy of the beam is localized over a ring, i.e. OAM beams, the aperture size shall be larger than D plus the vortex beam's diameter.

Although, by design, we limited our TAM plates to create two distinct OAM modes along the z-direction, it is possible to generate more OAM modes within the longitudinal range, L. To achieve this two conditions must be fulfilled: a) the metasurface diameter should satisfy Eq. (8) to ensure a sufficient extent for the beam, b) a wide range of cone angles (spatial frequencies) should be supported by the metasurface to constructively interfere each OAM mode over a short focal depth, thus allowing several OAM modes to constructed along the optical path. This is analogous to how a Fourier series can construct sharp edges by adding harmonics with high frequency. The largest spatial frequency is limited by the metasurface resolution; where $k_\rho|_{\text{max}} \sim 1/\partial r$, ∂r being the unit cell separation (which is less than 1 μ m for our metasurface scheme). By fulfilling a) and b) more than two OAM modes can be cascaded along the optical path. As suggested by Eq. (8), the aperture size scales linearly with the longitudinal extent of the beam, L.

Supplementary Note 4: Polarization-Sensitive Vortex

As alluded to in Section 2 in the main text, a longitudinally-varying vortex can be constructed by cascading two (or more) OAM series $\tilde{\psi}^{\ell}$ along the optical path. By allowing $\tilde{F}^{\ell}$ to take the form of a polarizer, the generated vortex becomes sensitive to incident polarization.

To illustrate this, here we construct a device implementing the matrix-valued vortex $\tilde{\Psi}^{\ell_1 \to \ell_2} = \tilde{\psi}^1 + \tilde{\psi}^2$, setting $\tilde{F}^1$ and $\tilde{F}^2$ as linear analyzers for x-polarization over the space regions $\{8\,\mathrm{mm} \leq z \leq 16\,\mathrm{mm}\}$, and $\{16\,\mathrm{mm} \leq z \leq 24\,\mathrm{mm}\}$, respectively. A device implementing the profile $\tilde{\Psi}^{\ell_1 \to \ell_2}$ will respond to x-polarized light, preferentially, by producing a vortex beam that changes the helicity of its twisted wavefront, signified by ℓ, from ℓ_1 to ℓ_2, with propagation.

The measured and simulated response of the device are shown in Supplementary Fig. 2 under three input polarizations: $0°$, $45°$, and $90°$. The measured transverse and longitudinal profiles of the beam show that the effective topological charge evolves from $\ell = 1$ to $\ell = 2$ with propagation along z, signified by the increase in the beam's diameter, and confirmed with the interferometric measurements with a tilted Gaussian beam. The phase dislocations (singularities) in the wavefront, i.e. the topological charges, are marked by red circles on the fork-like fringes in the insets of Supplementary Fig. 2(a), in agreement with the designed ℓ-value in each region. Additionally, this OAM plate is polarization-sensitive; it generates a spatially-varying vortex with maximum intensity in response to incident x-polarization, as designed, and responds to other polarizations (for e.g., $45°$) with reduced output intensity, while completely extinguishing the orthogonal y-polarization, in agreement with the theoretical prediction in Supplementary Figs. 2(c-d) and Malus's law. Importantly, this diattenuation behavior, typically realized through absorptive polarizers, is achieved here using a unitary (lossless) metasurface by means of interference. In essence, the higher diffraction orders at the Fourier plane of the metasurface, which were filtered away, act as channels that preserve energy conservation. These diffraction orders represent the fundamental trade-off, inherent in our dual-matrix holography implementation, to realize phase and amplitude modulation with a lossless platform.

Supplementary Note 5: Angular Momentum Density Calculation

TAM plates can generate optical vortices with a topological charge and polarization that vary with propagation in space. This evolution occurs locally at the center of the beam through a deliberate exchange of energy and momentum with its side lobes (outer rings). Here, we outline our method for calculating the angular momentum density (spin and orbital). Integrating these quantities over a cross section of the output beam, enclosing its diameter, confirms that both the SAM and OAM are globally conserved at each propagation distance, as depicted in Supplementary Fig. 4. Note that the TAM plates discussed in this work operate in the paraxial regime where a) the longitudinal components of the electric field can be neglected, and b) the SAM and OAM are additive and decoupled.

OAM density We start by deriving an expression for the OAM density following Refs. [6,7] in which the time-averaged Poynting vector is given by

$$\mathbf{P} = c^2\epsilon_0\frac{1}{2}\Re\{\mathbf{E}\times\mathbf{B}^*\}, \tag{9}$$

the magnetic flux density, $\mathbf{B}$, can be expressed in terms of the electric field by making use of Maxwell-Faraday equation ($\nabla\times\mathbf{E} = -i\omega\mathbf{B}$). The Poynting vector then becomes

$$\mathbf{P} = \frac{\epsilon_0 c^2}{2\omega}\Re\{i\mathbf{E}\times(\nabla\times\mathbf{E})^*\}. \tag{10}$$

Here, ϵ_0 is the free space permittivity (8.854×10^{-12} F/m), ω is the angular frequency, and c is the speed of light in vacuum. The OAM density is then evaluated from

$$\mathbf{j} = \frac{1}{c^2}(\mathbf{r}\times\mathbf{P}), \tag{11}$$

where $\mathbf{r}$ is the position vector ($\mathbf{r} = \mathrm{x}\hat{x} + \mathrm{y}\hat{y} + \mathrm{z}\hat{z}$). The longitudinal component of OAM density ($\mathbf{j}_z$) is the quantity that is relevant to our purposes. Integrating $\mathbf{j}_z$ over a given transverse cross section of the beam yields the OAM associated with that area, denoted as $\mathbf{L}_z$ such that

$$\mathbf{L}_z = \int\int \mathbf{j}_z r dr d\phi. \tag{12}$$

Here, it is understood that $\mathbf{L}_z$ is evaluated per unit length.

SAM density In order to investigate the conservation of SAM one has to integrate the SAM density ($\mathbf{s}$) across the beam's transverse section and ensure that the global momentum remains constant at each z-plane. The SAM density distribution is expressed as [8,9]

$$\mathbf{s} = \frac{\epsilon_0}{2i\omega}[\mathbf{E}^*\times\mathbf{E}], \tag{13}$$

by integrating over the transverse cross section, the SAM (per unit length) can be obtained from

$$\mathbf{S} = \frac{\epsilon_0}{2i\omega}\int\int[\mathbf{E}^*\times\mathbf{E}] r dr d\phi. \tag{14}$$

In Supplementary Fig. 4 , we evaluated the quantities $\mathbf{L}_z$ and $\mathbf{S}$ from Eqs. (12) and (14) considering two different integration limits: a) small aperture of 100 μm taken around the beam's center, and b) a large aperture of 500 μm enclosing the side lobes of the beam. We refer to these integrated values as local and global OAM/SAM, respectively. One can take a step further and normalize $\mathbf{L}_z$ and $\mathbf{S}$ by the total energy of the beam. In this case, the energy density is readily obtained from the Poynting vector; $w = c\epsilon_0\frac{1}{2}\Re\{E\times B^*\}$. The total energy per unit length, $\mathbf{W}$, is then obtained by

integrating w over the transverse profile of the beam such that

$$\mathbf{W} = c\epsilon_0 \frac{1}{2} \int \int \Re\{E \times B^*\} r dr d\phi. \tag{15}$$

Normalizing $\mathbf{L}_z$ and $\mathbf{S}$ by $\mathbf{W}$ yields a quantity that is proportional to the mean OAM/SAM per photon, scaled by $1/(\hbar\omega)$; see for e.g., Eqs. (2.8) and (2.18) in Ref. [10]. In Supplementary Fig. 4, we directly investigated the absolute quantities $\mathbf{L}_z$ and $\mathbf{S}$ without normalization. We conclude that while $\mathbf{L}_z$ and $\mathbf{S}$ can vary locally, these quantities must be globally conserved, independently, at each propagation distance.

Supplementary Figure 1

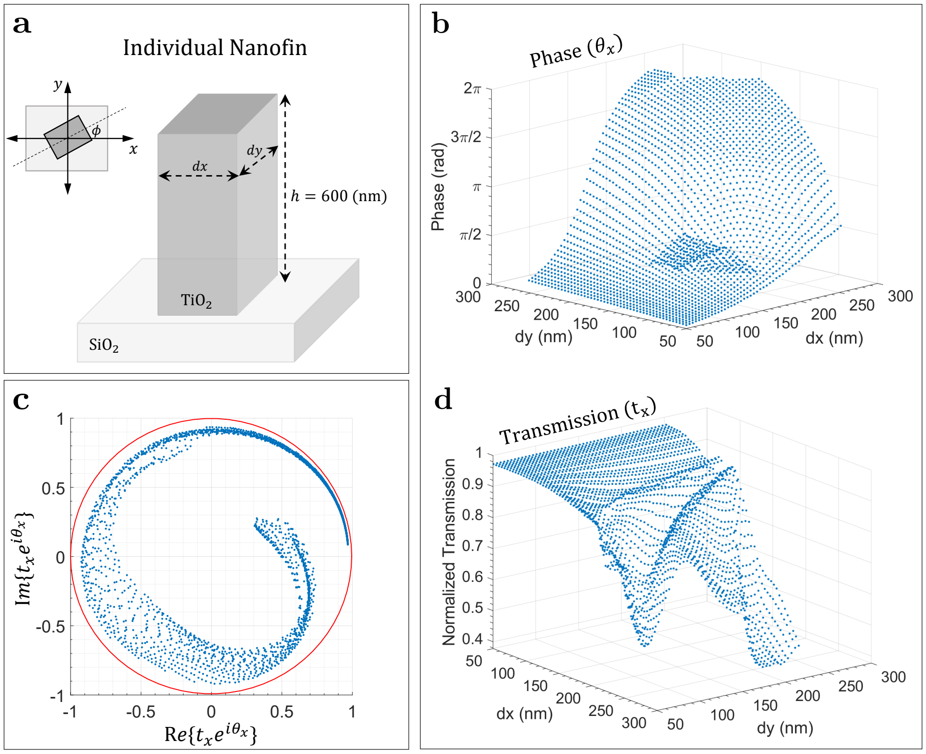

Supplementary Figure 1: Metasurface Design. **(a)** Schematic of the metasurface unit cell composed of a rectangular nanofin made of Titanium Dioxde (TiO_2) with a fixed height of 600 nm on top of a glass substrate[11]. **(b)** Phase response of the nanofins (in Radians) as a function of the transverse dimensions, d_x and d_y, for incident x-polarization. Each blue dot corresponds to a specific nanofin geometry. **(c)** The electric field amplitude transmission $t_x e^{i\theta_x}$ plotted on the complex plane for each of the 2500 individual geometries (blue dots). The red circle is the unit circle. **(d)** Normalized power transmission of the nanofins as a function of d_x and d_y, for input x-polarization. From the symmetry, the nanofin response for input y-polarization is readily obtained by swapping x and y in (b) and (d).

Supplementary Figure 2

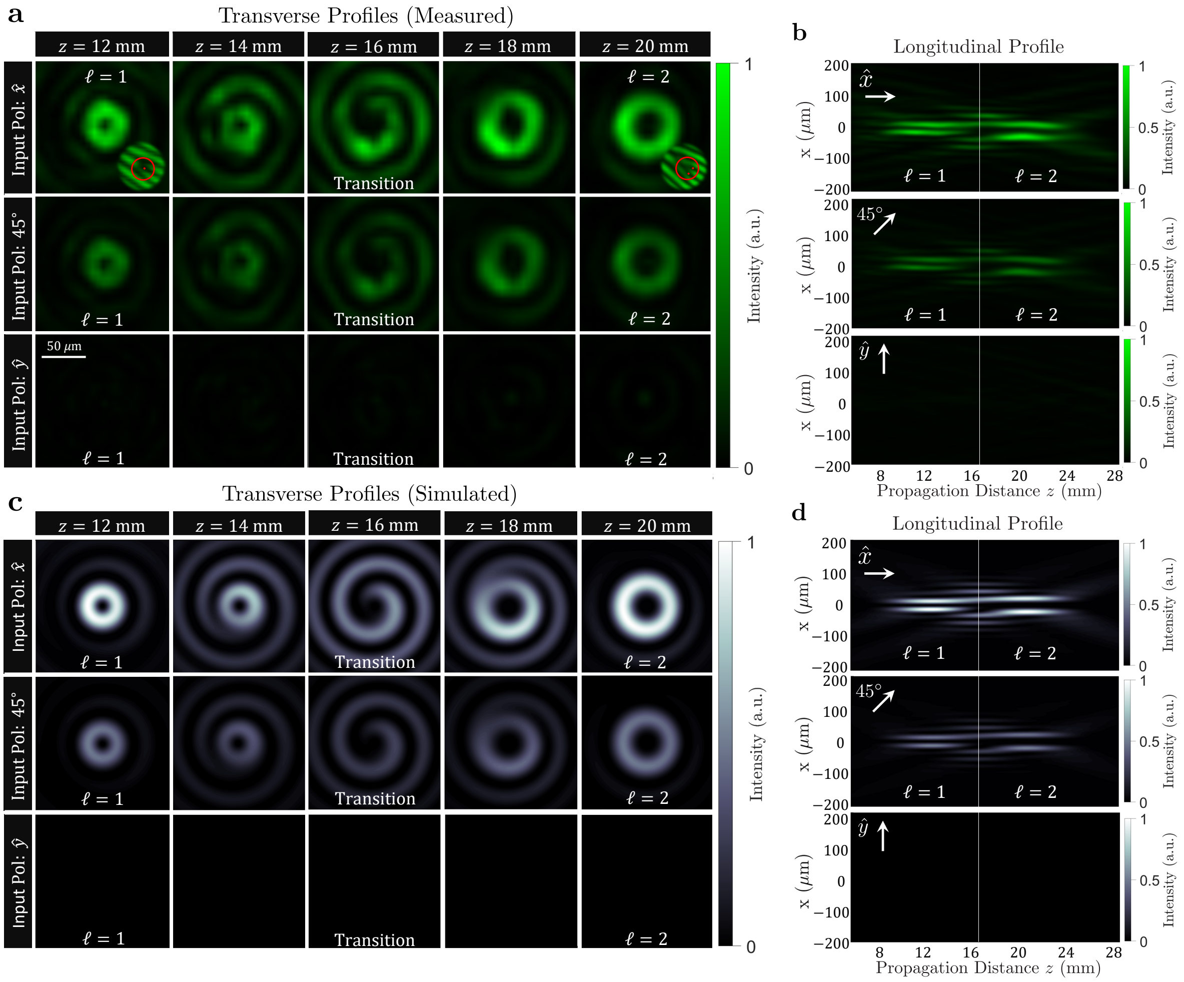

Supplementary Figure 2: Polarization-sensitive and longitudinally-varying optical vortex. **(a)** Measured transverse intensity profiles of a polarization-dependent vortex, $\tilde{\Psi}^{1\to 2}$, at multiple z-planes, exhibiting a topological transition from $\ell = 1$ to $\ell = 2$ with propagation. The red circles in the insets mark the underlying phase dislocations (topological charge) which were detected via an interferometric measurement with a tilted Gaussian beam. The target polarization function for this device takes the form of a linear x-polarizer which, preferentially, responds to $\hat{x}$ incident polarization with maximum intensity while extinguishing y-polarized light in accordance with Malus' law. **(b)** Measured longitudinal profile of the output beam from the same device, in response to rotating the incident polarization from $\hat{x}$ to $\hat{y}$, confirming its polarization-dependent behavior. **(c-d)** Simulated data corresponding to the measurements in (a-b) and showing very good agreement.

Supplementary Figure 3

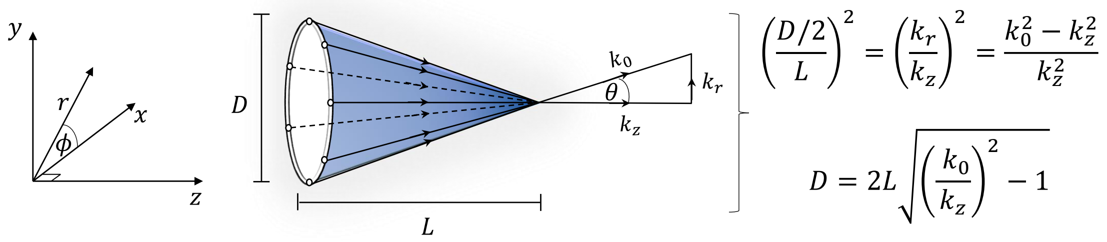

Supplementary Figure 3: Plane-wave expansion of a Bessel beam. The longitudinal and transverse wavenumbers, k_z and k_r, lie on the sides of a right angle triangle and are proportional to L and $D/2$, respectively.

Supplementary Figure 4

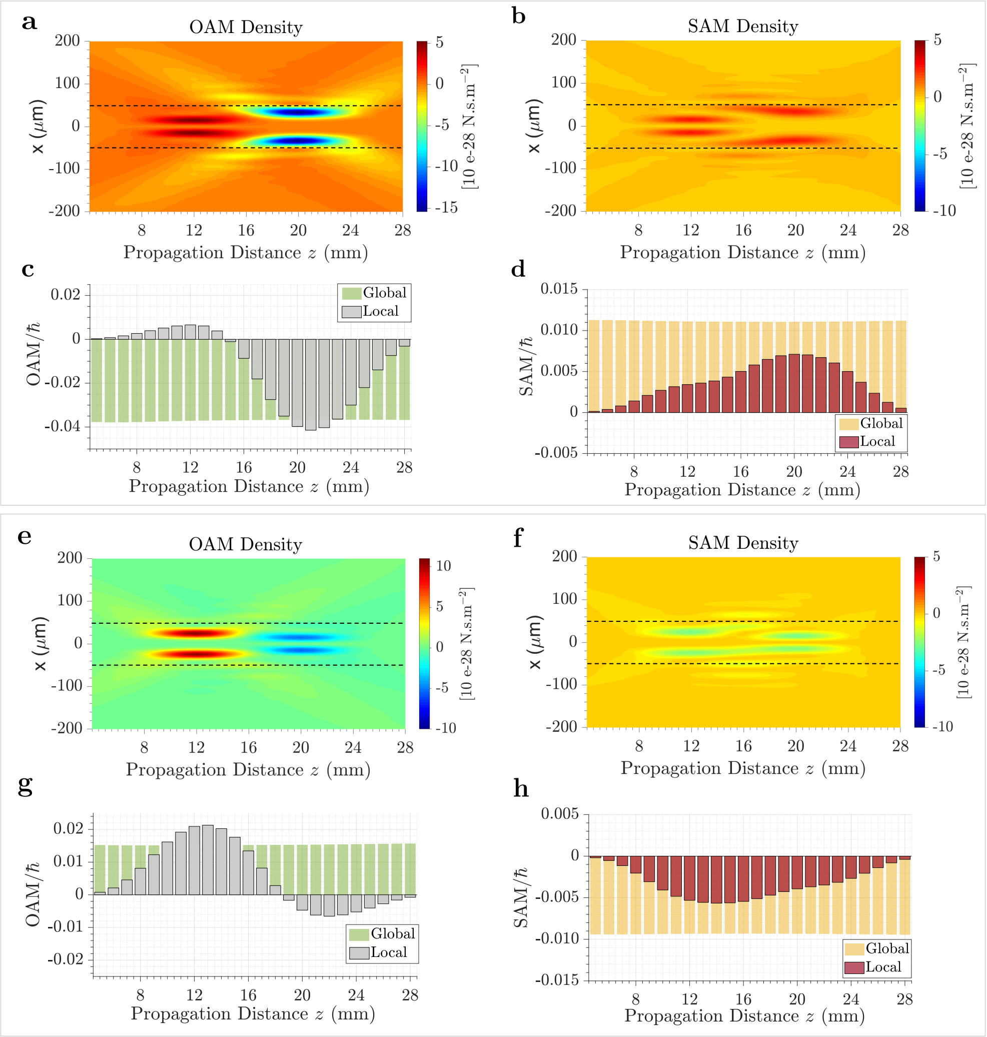

Supplementary Figure 4: Conservation of angular momentum. Calculated **(a)** OAM density and **(b)** SAM density of the output vortex beam $\Psi^{1\to-3}$ of Fig. 3 in the main article when the polarization state $|\lambda^{+}\rangle$ is incident on the device. While the OAM density evolves from a positive to a negative value with with propagation, the SAM density remains positive. **(c)** OAM obtained by integrating the OAM density in **(a)** over a small aperture (100 μm), denoted by the dashed lines in **(a)**, and large aperture (500 μm) across the transverse cross section of the beam, at each z-plane. We denote these quantities as local and global OAM, respectively. Similarly, **(d)** SAM obtained by integrating the SAM density in **(b)** over 100 μm and 500 μm at several propagation distances. While OAM and SAM can vary locally, their global quantities are independently conserved across the beam. **(e-f)** OAM and SAM calculations similar to those performed in **(a-d)** but for the case when the same polarization-switchable device is illuminated by the orthogonal polarization $|\lambda^{-}\rangle$. In this case, the generated vortex, $\Psi^{2\to-1}$, exhibits a transition in its OAM density from a positive to a negative value, while maintaining its SAM density at a negative value. **(g-h)** Local and global OAM and SAM confirming that the global angular momentum is always conserved.

Supplementary Figure 5

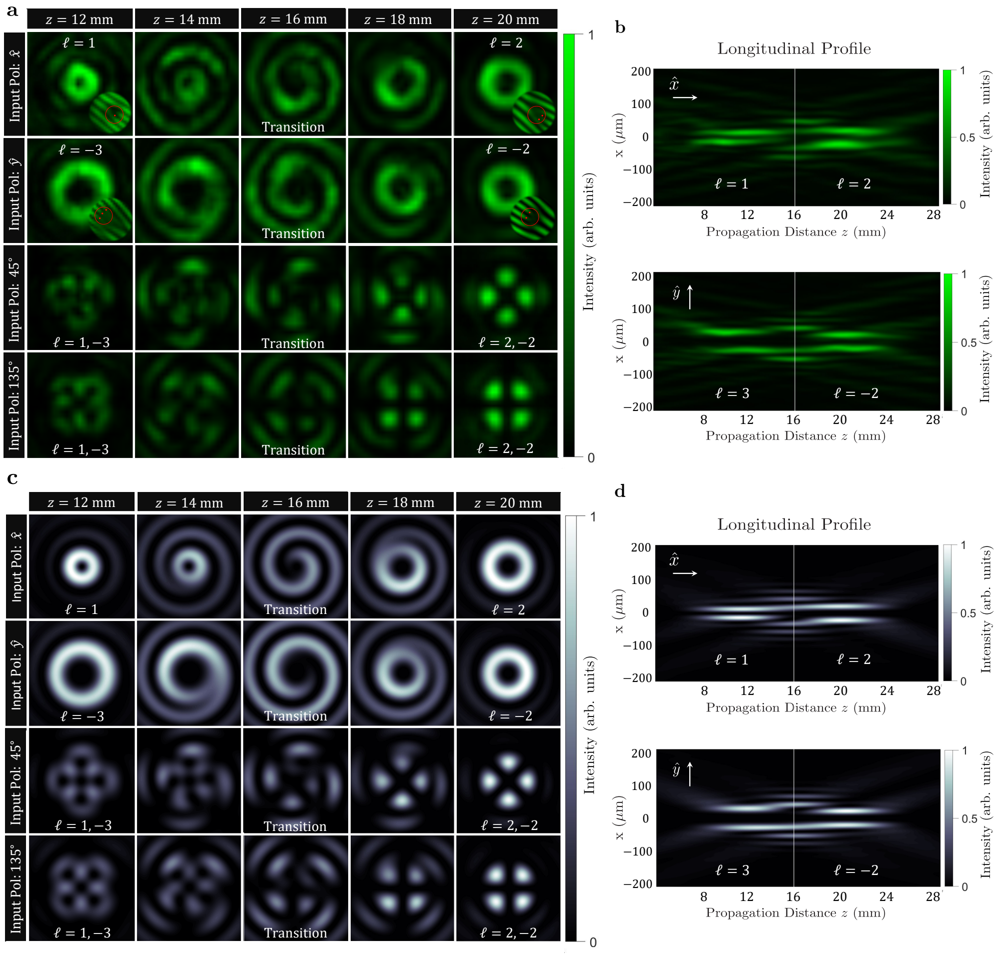

Supplementary Figure 5: Polarization-switchable TAM plate with linear eigen-polarizations. **(a)** Measured transverse intensity profiles of the output vortices in response to $\hat{x}$ and $\hat{y}$ polarizations, the eigen-polarizations of the device, in addition to an equally weighted average of the two: $45°$ and $135°$. Under x-polarized illumination, the generated vortex beam changes it topological charge from $\ell = 1$ to $\ell = 2$ as it propagates along the z-direction, whereas for the orthogonal polarization, $\hat{y}$, a different vortex evolving from $\ell = -3$ to $\ell = -2$ is produced. The topological charges have been quantified by detecting the number of phase dislocations in the wavefront via an interferomteric measurement with a tilted Gaussian beam. These phase singularities translate to discontinuities in the fork-like interference patterns as denoted by the red dots in the insets. At each z-plane, a weighted superposition of the two OAM states can be generated by rotating the incident polarization, as seen by the data for $45°$ and $135°$ incident polarizations, which depict the 4-petal-like structures. These interference patterns were obtained by placing an analyzer, oriented along the incident polarization, before the CCD camera; essentially projecting the output OAM states, which are orthogonally polarized, onto a common polarization bases (otherwise, the two output OAM states will not interfere). **(b)** Measured longitudinal intensity profile at the output of the device under two orthogonal incident polarizations, $\hat{x}$ and $\hat{y}$, confirming the generation of two distinct vortices with spatially-varying topological charges. **(c-d)** Simulated data corresponding to (and in full agreement with) the result in (a-b).

Supplementary Figure 6

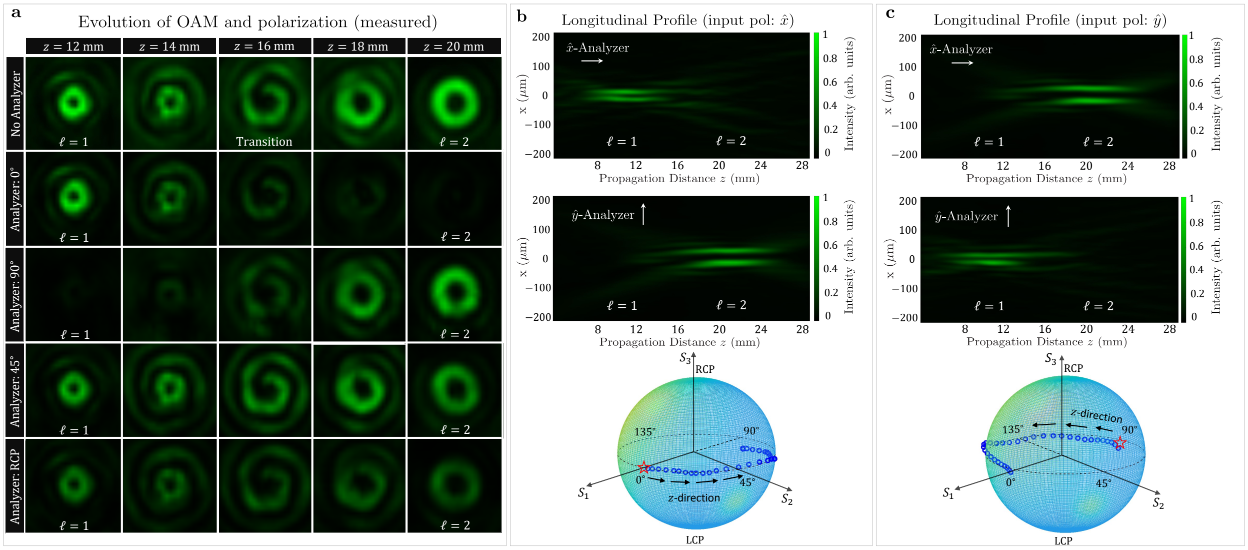

Supplementary Figure 6: Evolution of polarization and OAM of the vortex beam produced from the TAM plate depicted in Fig. 5 (main text). The output beam changes its topological charge and rotates its polarization state as a function of the propagation distance. **(a)** Measured transverse intensity profiles of the generated vortex, in response to incident $\hat{x}$-polarization, with no analyzer and after passing through an analyzer oriented at 0°, 90°, 45°, and circular polarization analyzer. As the beam propagates, it changes its topological charge value from $\ell = 1$ to $\ell = 2$ and rotates its polarization from $\hat{x}$ to $\hat{y}$ polarization. **(b)** Measured longitudinal intensity profile at the output of the device after passing through an analyzer oriented at 0° and 90° with respect to the horizontal axis, complementing the result in **(a)**. The adiabatic evolution of the polarization state, from $\hat{x}$ to $\hat{y}$ polarization, obtained by performing full Stokes measurement, is illustrated on the Poincaré sphere as a function of the propagation distance, z. The starting point of the trajectory is denoted by the red mark. **(c)** Longitudinal intensity profile and polarization state trajectory, similar to **(b)**, but under incident $\hat{y}$ polarization, confirming the generic HWP functionality of our device regardless of the incident polarization. All colorbars refer to the normalized intensity in arbitrary units.

Supplementary Figure 7

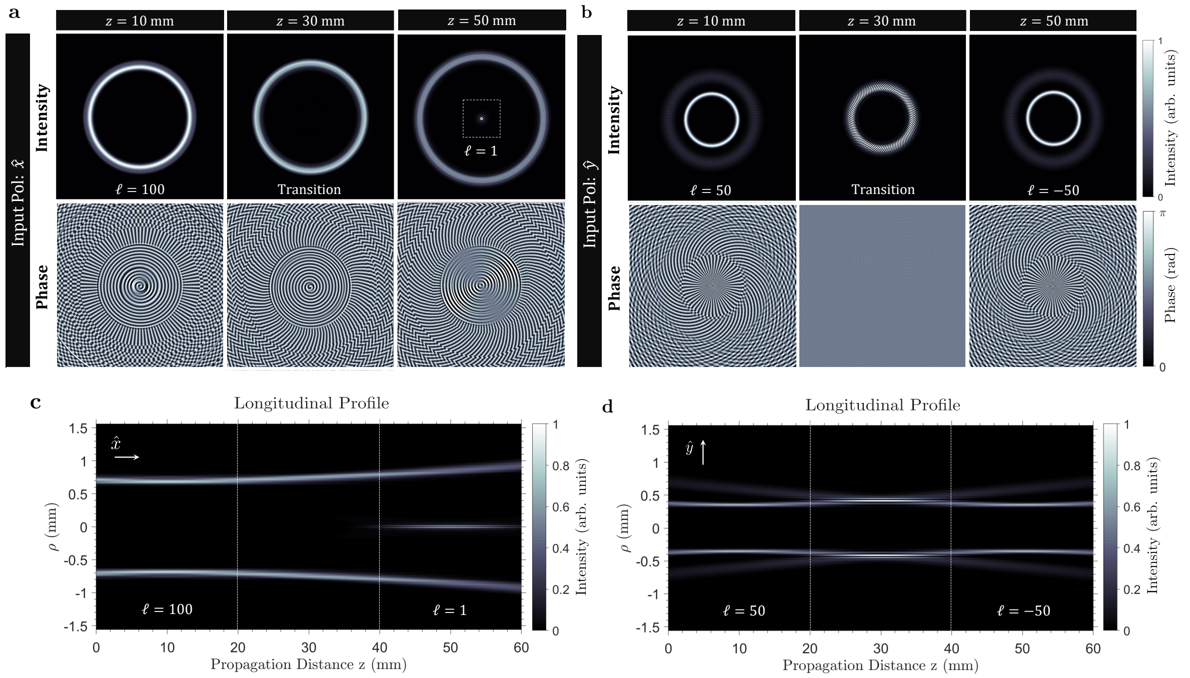

Supplementary Figure 7: Polarization-switchable TAM plate for generating vortex beams with large differential OAM values ($\Delta\ell$). (a-b) Simulated transverse intensity and phase profiles of the output vortices in response to $\hat{x}$ and $\hat{y}$ polarizations, i.e. the eigen-polarizations of the device. Under x-polarized illumination, the device generates vortex beam $\Psi^{100\to1}$, evolving from $\ell = 100$ to $\ell = 1$ as it propagates along the z-direction, whereas for the orthogonal polarization, $\hat{y}$, the same device generates the vortex beam $\Psi^{50\to-50}$, thus reversing its handedness with propagation. Notice how the wavefront unwraps its helicity at $z = 30$ mm, becoming nearly flat, as the beam reverses its topology. **(c-d)** Longitudinal intensity profiles corresponding to the beams in (a-b). Here, $\Psi^{100\to1} = \psi^{100} + \psi^{1}$, designed such that ψ^{100} and ψ^{1} contribute to the beam (constructively interfere) over the space regions $\{0\,\text{mm} \leq z \leq 20\,\text{mm}\}$, and $\{40\,\text{mm} \leq z \leq 60\,\text{mm}\}$, respectively. This ensures a sufficient distance for ψ^{100}, $\{20\,\text{mm} \leq z \leq 40\,\text{mm}\}$, to disperse its energy into its outer rings before the transition to $\ell = 1$ occurs. Similarly, $\Psi^{50\to-50} = \psi^{50} + \psi^{-50}$ where ψ^{50} and ψ^{-50} are designed to occur at the center of the beam over the same regions. At each z-plane, the global angular momentum is conserved. To implement this TAM plate, the diameter of the metasurface shall be large enough to ensure that the large OAM modes are maintained over the desired propagation distance L while overcoming diffraction, as discussed in Supplementary Note 3. Here, the aperture size (metasurface diameter) was chosen as $D = 2.3$ mm which was validated by running Kirchhoff's diffraction simulations, showing indeed quasi diffraction-less propagation over the desired range, L. Spatial dynamics of these beams can be found in Supplementary Movies 3 and 4.

Supplementary References

1. Dorrah, A. H., Rubin, N. A., Zaidi, A., Tamagnone, M. & Capasso, F. Metasurface optics for on-demand polarization transformations along the optical path. *Nature Photonics* **15**, 287–296 (2021).

2. Mendoza-Yero, O., Mínguez-Vega, G. & Lancis, J. Encoding complex fields by using a phase-only optical element. *Opt. Lett.* **39**, 1740–1743 (2014).

3. Balthasar Mueller, J. P., Rubin, N. A., Devlin, R. C., Groever, B. & Capasso, F. Metasurface polarization optics: Independent phase control of arbitrary orthogonal states of polarization. *Phys. Rev. Lett.* **118**, 113901 (2017).

4. McGloin, D. & Dholakia, K. Bessel beams: Diffraction in a new light. *Contemporary Physics* **46**, 15–28 (2005).

5. Born, M. & Wolf, E. *Principles of Optics (Sixth Edition): Chapter VIII - Elements of the theory of diffraction*, 370–458 (Pergamon, 1980).

6. Litvin, I. A., Dudley, A. & Forbes, A. Poynting vector and orbital angular momentum density of superpositions of bessel beams. *Opt. Express* **19**, 16760–16771 (2011).

7. Schulze, C., Dudley, A., Flamm, D., Duparré, M. & Forbes, A. Measurement of the orbital angular momentum density of light by modal decomposition. *New Journal of Physics* **15**, 073025 (2013).

8. Belinfante, F. On the current and the density of the electric charge, the energy, the linear momentum and the angular momentum of arbitrary fields. *Physica* **7**, 449 – 474 (1940).

9. Humblet, J. Sur le moment d'impulsion d'une onde électromagnétique. *Physica* **10**, 585 – 603 (1943).

10. Allen, L., Padgett, M. J. & Babiker, M. IV The orbital angular momentum of light. In *Progress in Optics* ***39***, 291–372 (Elsevier, 1999).

11. Devlin, R. C., Khorasaninejad, M., Chen, W. T., Oh, J. & Capasso, F. Broadband high-efficiency dielectric metasurfaces for the visible spectrum. *Proceedings of the National Academy of Sciences* **113**, 10473–10478 (2016).
