# Supplementary material for: Structuring total angular momentum of light along the propagation direction with polarization-controlled meta-optics
Source: Nat Commun. 2021 Oct 29;12:6249. doi: 10.1038/s41467-021-26253-4 (PMC8556329; doi:10.1038/s41467-021-26253-4)
Supplement: Supplementary file 2 — Description of Additional Supplementary Files [file 41467_2021_26253_MOESM2_ESM.pdf]

Description of Additional Supplementary Files

File name: Supplementary Movie 1
Description: Measured transverse intensity profiles of a vortex beam changing its topological charge value from 1 to -3 as it propagates away from the metasurface.

File name: Supplementary Movie 2
Description: Measured transverse intensity profiles of a vortex beam changing its topological charge value from 2 to -1 as it propagates away from the metasurface.

File name: Supplementary Movie 3
Description: Simulated spatial evolution of a vortex beam reducing its topological charge from 100 to 1 as a function of propagation distance.

File name: Supplementary Movie 4
Description: Simulated spatial evolution of a vortex beam reversing its topological charge from 50 to -50 as a function of propagation distance.
